# Supplementary material for: Input–output maps are strongly biased towards simple outputs
Source: Nat Commun. 2018 Feb 22;9:761. doi: 10.1038/s41467-018-03101-6 (PMC5823903; doi:10.1038/s41467-018-03101-6)
Supplement: Supplementary file 1 — Supplementary Information [file 41467_2018_3101_MOESM1_ESM.pdf]

SUPPLEMENTARY NOTE 1

Algorithmic Information Theory (AIT)

In this section we briefly provide some basic notation, definitions, and key results of algorithmic information theory (AIT) that are used in the main text and in further appendices. Fuller descriptions can be found in ref. 1, which is a standard reference for AIT. Other good introductions can be found for example in refs. 2 and 3.

Basic notation and definitions

Notation

Throughout this text, the length of a binary string x is denoted as $l(x)$. The set of all binary strings x of length $l(x) = n$ is denoted by $\{0,1\}^n$; the set of all binary strings of length $\leq n$ is written as $\{0,1\}^{\leq n}$; the set of all possible binary strings is denoted $\{0,1\}^*$. For a string x, x^n represents n concatenated copies of the string x. For example, $0^4 = 0000$.

We follow standard usage of the notation $\mathcal{O}(1)$ as in AIT and physics[4]: For a function $\zeta(u)$ we write $\zeta(u) = \gamma(u) + \mathcal{O}(1)$ if for all u, $|\zeta(u) - \gamma(u)| < M$, for some constant M. For example, if $f(u) = u + 2$ or $f(u) = \sin(u) + u$, then we can write $f(u) = u + \mathcal{O}(1)$.

Universal Turing Machines

Turing machines are abstract generic digital computation devices proposed in 1936 by Alan Turing[5]. A *universal Turing machine* (UTM) is a Turing machine that can simulate the behaviour of any other Turing machine, and a programming language that can be used to implement a UTM is called *Turing complete.* Most commonly used programming languages are Turing complete.

One reason that UTMs are important is because of a hypothesis known as the *Church-Turing thesis* which states that any effectively computable function can, in principle, be calculated by a UTM. This thesis, although not formally proven, is widely believed to be true.

Interestingly, Turing's motivation for inventing UTMs was to prove that there are calculations a computer cannot do. If a program running on a UTM stops and presents an output after a finite number of steps, the program is said to *halt.* Turing famously showed that there is no universal way to decide whether any given arbitrary program will halt or not. This principle, which has deep implications for the philosophy of mathematics and computer science, is called the *halting problem.*

It is customary in AIT to prove results for binary strings, a convention we will follow below. This convention can be understood intuitively by recognising that in principle any discrete object can be reduced to a binary string by a standard indexing procedure. Also, since a UTM can simulate any other UTM, and UTMs can be defined that take binary strings as their input, there is no loss of generality in this convention.

Proving that a given map is Turing complete is often not straightforward. However one simple test for proving that a map is *not* Turing complete is to establish that all input programs halt. A *computable* map (or function) is one that produces an output and then halts for all valid inputs. By this criterion, we see that many maps used in science and engineering are computable; for example, any RNA nucleotide sequence we present to the Vienna computational folding package[6] will adopt *some* secondary structure (i.e. output), and not keep running indefinitely. Nevertheless, there are important examples of physical systems that can be mapped onto UTMs, and thus have properties that are undecidable because they are equivalent to solving the halting problem[7,8]. But these will not concern us here.

Basics of Kolmogorov-Chaitin complexity

Definition of Kolmogorov-Chaitin complexity

The historical development of AIT by Solomonoff[9], Kolmogorov[10] and Chaitin[11,12] was motivated by attempts to quantify the information content or randomness of individual objects such as binary strings or discrete geometries. In this way, AIT contrasts with the better known Shannon information theory which focuses on distributions instead of on individual objects. For example, even if individual strings in a distribution vary enormously in their complexity,

Shannon information only picks up the frequency with which each string appears, which may be unrelated to the complexity of an individual string. AIT does not measure the properties of the distribution, but instead quantifies the complexity of an individual string. Nevertheless, there are important relationships between AIT and Shannon information theory, see e.g. the standard references cited above or ref. 13 for an overview.

The fundamental insight of AIT is that the information content of an object can be defined algorithmically, by the minimum amount of information needed to describe or generate that object. Consider for example the following binary strings

$$(01)^n = 0101010101010101010101 ...$$
$$rand(1:n) = 1001110101011100010111111 ...$$

The first is intuitively 'simple' as the whole string can be described as "print '01' n times". Since the string conforms to a simple rule, and thus also a short description, the string is deemed simple in AIT. The second string is a typical randomly generated bit-string. Assuming that there are no hidden patterns in this string and that it does not conform to any simple rules or short description, then the shortest description may simply be to print the string in full. Hence it is deemed complex.

AIT characterises the information content, or *Kolmogorov-Chaitin complexity*, of an output x as the length of the shortest computer program that can generate x. More formally, the (plain) Kolmogorov-Chaitin complexity or simply Kolmogorov complexity $C_U(x)$ of a binary string x is defined as

$$C_U(x) = \min_q \{l(q) : U(q) = x\} \quad (1)$$

where $l(q)$ is the length of a binary program q in bits, and U is a UTM. That is, $C_U(x)$ is the length of the shortest program over all programs that print x and then halt. It is well known that $C_U(x)$ is *uncomputable*, meaning that even in principle there cannot exist a general algorithm for finding the exact value of $C_U(x)$, given x. This is established by reducing the problem of calculating $C_U(x)$ to the *halting problem.*

The invariance of complexity

The *invariance theorem* is a cornerstone of AIT. It states that $C_U(x)$ only depends on the choice of the UTM U up to an additive constant. That is, if U and V are both UTMs, then

$$|C_U(x) - C_V(x)| \leq c \quad (2)$$

for any x, where c is a constant independent of x, but depending on the choice of U and V. Intuitively, since one can always write a compiler to transform U to V, the constant c is smaller or equal to the size of the compiler. In the limit of large complexities this difference can be ignored, or alternatively, one can simply say that the Kolmogorov complexity is only defined up to a constant. Hence the subscript is dropped and we speak of 'the' Kolmogorov complexity $C(x)$.

A few quick notes. Firstly, $C(x)$ is always defined with respect to a particular UTM. The invariance theorem simply tells us that up to a constant, it does not matter what UTM is used. Secondly, the invariance theorem also does not mean that Kolmogorov complexity is the shortest description of a string x for any UTM. For example, it is always possible to define a UTM that has a particular string x stored, so that $C(x)$ is effectively zero for that string. It is worth quoting ref. 1 (pp 106) on this topic: *The key point is not that the universal description method necessarily gives the shortest description length in each case, but that no other description method can improve on it infinitely often by more than a fixed constant.*

Most strings are complex

Most binary strings have a Kolmogorov complexity close to their length in bits. This follows from the following simple counting argument: for a given number of bits n, there are 2^n different strings in $\{0,1\}^n$, and $2^n - 1$ shorter strings in the set $\{0,1\}^{\leq(n-1)}$. There are enough strings that any of the 2^n strings in the former set might be compressed to one in the latter set. However, 2^{n-1} of these strings are are just one bit shorter, so at best, 50% of the strings in $\{0,1\}^n$ can be compressed by at most one bit. More generally, at most a fraction of 2^{-k} of strings in $\{0,1\}^n$ are compressible by exactly k digits, because there are only 2^{n-k} binary strings of that length available. More generally, the fraction of strings of length n that can be compressed by at least k digits is $2^{-k+1} - 2^{-n}$. For example, for strings of length $n = 100$, only about 0.2% can be compressed by $k = 10$ bits or more. In other words, 99.8% of

$n = 100$ strings cannot be compressed to less than 90% of their length. A string is considered to be *algorithmically random* if it cannot be compressed by more than a few bits, and as seen in this example, most strings can only be compressed by a relatively small amount. Therefore, a randomly chosen string is likely to be algorithmically random.

Prefix complexity

For technical reasons, as emphasised by Chaitin, it is often convenient to work with *prefix-codes* or *instantaneously decodable* codes for which the set of code words (i.e. programs) is prefix-free[1], that is, if q and r are both valid programs which can produce outputs, then in a prefix code q cannot form the first $l(q)$ bits (i.e. it cannot form a prefix) of program r, and vice versa. This implies that there is no need for a spacer or other symbol to mark the beginning and end of concatenated programs. Hence any string of bits can be unambiguously decoded into separate programs.

Having introduced the plain complexity $C(x)$, we now define the closely related *prefix complexity* $K_W(x)$ [11,14]:

$$K_W(x) = \min_q \{l(q) : W(q) = x\} \tag{3}$$

where W is a prefix UTM i.e. W is a *self-delimiting machine* where the programs are prefix-free. As above for the plain complexity $C(x)$, an invariance theorem holds for its prefix-free analogue, and so we can drop the subscript of W on $K_W(x)$. While $K(x)$ differs from $C(x)$ in important technical ways, quantitatively they are in fact very close, as $K(x)$ is also uncomputable, being equal to $C(x)$ up to a term logarithmic in $C(x)$: It is known[1] that

$$\begin{aligned} C(x) &\leq K(x) && (4)\\ &\leq C(x) + \log_2(C(x)) + 2\log_2\log_2(C(x)) + \mathcal{O}(1) && (5)\\ &\sim C(x) && (6) \end{aligned}$$

so that the two complexity measures are normally quite close to one another.

Conditional Kolmogorov complexity

In Shannon information theory, it is convenient to derive relationships between concepts such as joint, mutual or conditional entropies/information. For example, the conditional information $H(Y|X)$ is defined as the entropy in the variable Y given that the variable X takes a particular value $X = x$, averaged over all possible values of x. Somewhat analogous relationships can be derived for Kolmogorov complexity. However, in contrast to Shannon entropies, for which these relationships are statistical averages over distributions, for Kolmogorov complexity the relationships hold for individual objects. For example, a kind of mutual information can be defined as $I(x : y) = K(x) + K(y) - K(x, y)$ which measures the difference between generating x and y separately, and generating them jointly (measured by $K(x, y)$). Similarly, the conditional prefix Kolmogorov complexity $K(x|y)$ can be intuitively interpreted as the length of the shortest program that, when fed into a prefix UTM W, generates x and halts, if W is also given y "for free". If y is genuinely independent of x, i.e., roughly saying, if having y doesn't help W calculate x at all, then we expect that $K(x|y) = K(x) + \mathcal{O}(1)$, whereas if, say $y = 2x$, then it will be very easy for W to generate x and $K(x|y) = \mathcal{O}(1) \ll K(x)$. Note that there is a subtle difference between $K(x|y)$ and $K(x|y^*)$, with the latter defined as the conditional prefix Kolmogorov complexity given that the shortest *program* to calculate y is given.
We will use the following two relations:

$$K(x|y) \leq K(x) + \mathcal{O}(1) \tag{7}$$

$$K(x) \leq K(x|y) + K(y) + \mathcal{O}(1) \tag{8}$$

Intuitively, the first identity follows because adding information (y in this case) could mean you need less information to generate x, and if y is completely irrelevant then you can always generate x by ignoring y. Similarly, for the second identity, if the right side were less than $K(x)$, then it would represent a shorter way to generate x, which, by definition would define the true $K(x)$.

Universal probability and the coding theorem

For a prefix UTM, the probability of generating a particular input program of length l by random coin flips is 2^{-l}. The *universal probability*[9,14] of a string x is defined as

$$P_U(x) = \sum_{q:U(q)=x} 2^{-l(q)} \tag{9}$$

which is the probability that a prefix UTM U outputs x when fed with a random program q (e.g. generated by coin flips). So the universal probability simply sums over the probability of all possible programs that generate a given output x. One reason to use a prefix UTM is Kraft's inequality, which states that if $\mathcal{F}$ is a set of binary prefix-free code words, then $\sum_{f\in\mathcal{F}} 2^{-l(f)} \leq 1$ which ensures that $\sum_x P_U(x)$ converges. However, because of the halting problem, it is impossible to know in general if a given program q will halt. If a program does not halt, then it does not produce any output, and there would be some probability mass absorbed by these non-halting programs. Hence summing over halting programs would yield a mass of less than 1. Thus strictly $P_U(x)$ is a semi-measure; it can only be approximated from below, and $\sum_x P_U(x) \leq 1$.

The (algorithmic) *coding theorem* was established by Leonid Levin in 1974[14]. It connects $K(x)$ and the universal probability $P_U(x)$ as follows

$$2^{-K(x)} \leq P_U(x) \leq 2^{-K(x)+d} \tag{10}$$

where d is some constant independent of x, but possibly depending on the choice of UTM, U. The coding theorem can be expressed differently as

$$P_U(x) = 2^{-K(x)+\mathcal{O}(1)} \tag{11}$$

Because the invariance theorem implies that $P_U(x)$ is asymptotically independent of U, the subscript U is conventionally dropped, and instead one just writes $P(x)$. In essence this theorem says that the probability of a UTM printing x when fed with a random program is largely determined by the Kolmogorov complexity of x: Low complexity or 'simple' outputs are highly probable, while highly complexity outputs are exponentially less likely. The fact that the lower bound $P(x) \geq 2^{-K(x)}$ holds is clear, as the summation in equation (9) contains the term $2^{-K(x)}$. This lower bound was in fact pointed out earlier by Solomonoff[9], although he didn't use prefix machines. The contribution of Levin was to show that the upper bound $P(x) \leq 2^{-K(x)+d}$ also holds (for some constant d). This latter claim is neither obvious nor trivial in the UTM setting[1,14,15]. For example, for UTMs there are infinitely many possible programs, and so *a priori* we might expect that some high complexity outputs could nevertheless have high probability because many longer programs generate them. The coding theorem shows that this is not generally the case.

SUPPLEMENTARY NOTE 2

Upper bound on probability for computable maps

In the main text we apply the following upper bound for computable maps:

$$P(x) \leq 2^{-K(x|f,n)+\mathcal{O}(1)} \tag{12}$$

where $K(x|f,n)$ is the complexity of an output x, given the map f and given n, which parametrises the size of the input space I of the input-output map, e.g. for binary sequences of fixed length n, the size is 2^n possible inputs. This equation (or something similar to it) for the upper bound can be found in standard texts such as refs. 3 and 1, but we also provide a derivation here, following a standard method.

Consider the following algorithm A:

(i) Enumerate all inputs using n.
(ii) Map these inputs to their outputs, according to the rules specifying the map f.
(iii) Print the resulting list of each output x and its corresponding probability $P(x)$ (i.e. frequency in I).

Since f and n are given, the complexity of the algorithm is $K(A) = \mathcal{O}(1)$. This procedure is an example of a well known result of AIT, namely that enumerating all possible objects in a set can be algorithmically much simpler than

generating a typical element of the set. For example, the information required to construct $\{0,1\}^n$, the set of all binary strings of length n, is only about $\log_2(n) + \mathcal{O}(1)$ bits, and possibly much less for very simple n, whereas a typical member x of this set has close to maximal complexity, i.e. $K(x) = n + \mathcal{O}(1)$. Hence enumerating the whole set requires much less information than does specifically generating one typical member. In the same way, the procedure above to generate all outputs can require substantially less information than the information needed to generate a specific output x.

Now, it is well known from information theory[15] that given a discrete distribution, one can efficiently encode outputs using a Shannon-Fano-Elias (SFE) code, which consists of prefix-free code words $E(x)$ of length (in bits)

$$l(E(x)) = \left\lceil \log_2\left(\frac{1}{P(x)}\right)\right\rceil + 1 \tag{13}$$

where $\lceil\cdot\rceil$ denotes taking the integer part. In this manner, we have a method for assigning bit strings to outputs x. So, using a SFE code, and given f and n, we can describe any output x using $l(E(x)) + \mathcal{O}(1)$ bits, where the $\mathcal{O}(1)$ term accounts for the fixed program to generate the SFE code. Because Kolmogorov complexity gives the shortest possible description length (within $\mathcal{O}(1)$ terms) for a given UTM, we must have that $K(x|f,n)$, which is the information (in bits) required to specify a given output x, given the input-output map f and n, is no larger than the SFE code description just derived, i.e.

$$\begin{aligned} K(x|f,n) &\leq l(E(x)) + \mathcal{O}(1) \qquad (14)\\ &= \log_2\left(\frac{1}{P(x)}\right) + \mathcal{O}(1) \qquad (15)\\ \Rightarrow P(x) &\leq 2^{-K(x|f,n)+\mathcal{O}(1)} \qquad (16)\end{aligned}$$

Note that we are abusing notation slightly, since we have used the letter f to denote both the *function*, as well as to denote the *program* for implementing the function f. Similarly we have written n to denote both the number as well as a program to calculate it, although for most n the size of the programme n^* will be $\log n + \mathcal{O}(1)$ since most n are not compressible. So technically we should write $K(x|f^*, n^*)$, but for simplicity of notation we simply write $K(x|f,n)$.

SUPPLEMENTARY NOTE 3

Limited complexity maps

Equation (12) is a very general statement that applies to a wide range of functions f. However, the details of f can affect the output probabilities $P(x)$. For instance, if for a given output x and mapping function f one has $K(x|f,n) \ll K(x)$, so that $2^{-K(x|f,n)} \gg 2^{-K(x)}$, then x would have a much higher probability to appear than predicted by the coding theorem. In other words, in this case, even if one knows $K(x)$, it is necessary to know the details of the map f in order to make predictions about $P(x)$.

We will leave the case of making predictions about $P(x)$ for general maps f for future work. In this manuscript, we instead consider one important special case, namely maps of limited complexity, which we define as maps for which asymptotically, i.e. for large x, $K(f) + K(n) \ll K(x) + \mathcal{O}(1)$ holds. Using standard inequalities we can then show that

$$\left.\begin{aligned} K(x) &\leq K(x|f,n) + K(f) + K(n) + \mathcal{O}(1)\\ K(x|f,n) &\leq K(x) + \mathcal{O}(1)\\ K(f) + K(n) &\ll K(x) + \mathcal{O}(1)\end{aligned}\right\} \Rightarrow K(x) \approx K(x|f,n) + \mathcal{O}(1) \tag{17}$$

from which it follows that occurrences of $K(x|f,n) \ll K(x)$ are asymptotically negligible, and so the inequality (12) becomes

$$P(x) \lesssim 2^{-K(x)+\mathcal{O}(1)} \tag{18}$$

which is asymptotically independent of f and n. Of course f and n still define the set of x that are possible, but given an x, this inequality holds for the probability that it is generated upon uniform sampling of inputs. It is not hard to see that for limited complexity maps it is the case that $K(x) = K(\text{set of all inputs that generate x}) + \mathcal{O}(1)$, since the the set can generate x and given x the set can be generated by enumerating all inputs and checking those

that generate x. Thus, as in the main text, for limited complexity maps, the structural variation in outputs x is generated with as little artefactual biasing from the mapping rule-set as possible. If instead the map *itself* clearly determines many aspects of output structure irrespective of input choice, then any complexity in these aspects must be due to artefactual biasing of the map, and not the information contained in a given input. In the main text and in Supplementary Note 12 we show an explicit matrix based map which is generally not a limited complexity map, and therefore fails this "no artefactual biasing" test.

Our arguments above invoke $K(f) \ll K(x)$ and $K(n) \ll K(x)$ for a typical x. Most examples we explore are maps of fixed complexity $K(f) = \mathcal{O}(1)$, for which one can always find large enough x so that these inequalities clearly hold. Since $K(n)$ scales asymptotically as $\log(n)$ it would seem that simplicity bias should also hold for maps that scale as $\log(n)$, e.g. ones that are not necessarily fixed. On the other hand, if $K(f)$ scales linearly with n (which is how $K(x)$ scales) or more (e.g. in the matrix map $K(f) \sim \mathcal{O}(n^2)$) then the inequality won't hold. So exactly what maximum scaling of $K(f)$ with n is possible for the map f to still show simplicity bias phenomenology, and exactly how this works for maps that we coarse-grain, where n is less well defined, remain open questions for future investigations.

Another very interesting finding for the maps we study in this paper is that our simplicity bias predictions still work when x is small enough that we do not expect the $K(f) \ll K(x)$ to strictly hold. For an example, in Supplementary Note 9 we explore the effect of input length on the prediction of simplicity bias for the input-output map from an RNA sequence to its corresponding secondary structure. While $K(f)$ is not known for the Vienna package[16], the software package used here to obtain those secondary structures[6], $K(f)$ is likely to be greater than the small number of bits necessary to describe the 20 letter-long outputs from this map. And yet, we clearly observe simplicity bias for this map. On the other hand, in Supplementary Note 9 we show that for very short RNA strands, the simplicity bias predictions we make start to break down, as expected. Although the general arguments used to derive our upper bound can only be proven to hold in the limit of larger outputs x, we conjecture that these basic properties survive when moving out of this asymptotic regime and into regimes where $K(f) \ll K(x)$ may no longer strictly hold. It is not uncommon in physics and mathematics to find that an asymptotic law still works qualitatively outside of the domains for which it can be proven to hold. Something similar is likely to be at work here, meaning that our general predictions about simplicity bias have a wider domain of applicability than one might at first assume. Exactly how this works, and how it may depend on the scaling of $K(f)$ with n remain open questions for future work.

Finally, while we have mainly studied examples of fixed complexity maps, for the matrix maps with certain types of circulant matrices, described in Supplementary Note 12, we find very preliminary evidence that suggests that the simplicity bias holds not only for maps of fixed complexity, but also for maps where $K(f)$ grows asymptotically as $K(f) \sim \log(n)$, as we anticipated above. In the limit of large x the derivations above for equation (12) above still holds, but further investigation is necessary to work out more generally when and how simplicity bias depends on particular properties of the matrix map. It may be, for example, that it is most pronounced for the simpler fixed maps.

SUPPLEMENTARY NOTE 4

Limited complexity maps without simplicity bias

Linear maps cannot show bias

In the main text, we claim that for an input-output map to show simplicity bias, f must be a nonlinear function of its inputs. This is because linear transformations are not biased towards any outputs, as we show now.

If f were linear then its domain I is a discrete subset of a finite-dimensional space; f would also be bounded and continuous. This would imply that if two inputs are close in input space, the corresponding outputs would also be close to each other[17]. Likewise, any distance in input space will translate into a proportional distance in output space.

Moreover, while a linear transformation does not necessarily preserve angles between lines or distances between points, it does preserve ratios of distances between points lying on a straight line. Because of this property, if one were to select inputs on a grid, the ratios of the distances between the points on the grid would not be affected by the input-output map. The linear transformation would map the grid in input space to the grid in output space, and no point in output space would be "denser" - in terms of having more outputs in its neighbourhood - than any other point.

In a similar way, if instead of selecting inputs from a grid one were to sample them uniformly from a bounded subset of input space, this uniform distribution would still be present in output space, as the corresponding outputs would also be uniformly distributed. In summary, a linear map cannot produce bias towards any outputs. Therefore, for a map to show simplicity bias (or any kind of bias for that matter), it must be a nonlinear function of its inputs.

Simplicity bias is not a necessary consequence of conditions 1-5

In the main text we gave five conditions on maps which we conjecture are usually sufficient for observing simplicity bias. That is, we suggest that typical real-world maps satisfying these conditions will show simplicity bias.

However, it is important to point out that these five conditions do not in fact *necessitate* that the map will show bias. As a very simple counter example, consider a map which prints the first $n/2$ bits of n-bit inputs strings. This map has $N_I \gg N_O$, and the map is simple. Nevertheless, the output is a uniform distribution over $\{0,1\}^{n/2}$, and hence there is neither bias, nor simplicity bias. Of course, this projection map is a linear map, and so it does not satisfy the condition of linearity which we imposed. However, it could easily be altered slightly to make it nonlinear, while retaining a roughly uniform distribution. For example, we could alter the definition of the map to *'print 0^n if the sum of first $n/2$ digits is a prime number; otherwise print the first $n/2$ digits'*. This distribution would be biased toward the outputs $0^{n/2}$, but otherwise be uniform over the others.

The point of this perhaps rather artefactual example is to illustrate that one can create maps that satisfy our conditions, but do not display simplicity bias. Nevertheless, for all the real-word systems we examined in this work, we find that simplicity bias holds. So we conjecture that our five conditions are sufficient for most non-UTM maps that are generated from real-world systems . But this conjecture could be tested with further examples. Working out the exact formal necessary and sufficient requirements for simplicity bias in non-UTM contexts would form an interesting future project.

SUPPLEMENTARY NOTE 5

Estimating the range of $K(x|f,n)$

We will now estimate the range of values for $K(x|f,n)$ with $x \in O$. We begin with a lower bound on possible complexity values: Given f and n we can compute all the inputs, and produce all N_O outputs. Hence, we can describe any $x \in O$ by its index $1 \leq j \leq N_O$ in the set of outputs O. Therefore

$$K(x|f,n) \leq \log_2(j) + \mathcal{O}(\log_2(\log_2(j))) + \mathcal{O}(1) \tag{19}$$

where the second $\mathcal{O}(\log_2(\log_2(j)))$ term arises from the fact that the description is in prefix-free form. Since at least one output should have an index of $j = 1$, for that output all terms containing $\log_2(j)$ will be equal to zero, resulting in a lower bound for the range of $K(x|f,n)$:

$$\min_{x\in O}\left(K(x|f,n)\right) = \mathcal{O}(1) \tag{20}$$

Another slightly cruder way of estimating the minimum value of $K(x|f,n)$ follows from simply noting that the probability $P(x)$ should be always be less than one. In that case, our upper bound equation (12), implies that $\min\limits_{x\in O} K(x|f,n) \gtrsim 0$.

For an upper bound on $\max\limits_{x\in O} K(x|f,n)$, the indexing argument from equation (20) suggests that

$$\max_{x\in O}(K(x|f,n)) \leq \log_2(N_O) + \log_2(\log_2(N_O)) + \mathcal{O}(1) \tag{21}$$

It is possible to derive a similar upper bound using a standard AIT argument: if all strings can be used as programs that encode a map's outputs, then there are at most $\sum_{l=1}^{M} 2^l = 2^{M+1} - 1$ programs of length $l \leq M$. However, since we are using prefix codes, not all strings are available, and only roughly 2^M out of the set of all $2^{M+1} - 1$ programs can be used. For N_O outputs, this argument implies that one would need strings made of up to $\log(N_O)$ bits to encode all outputs, thus imposing an upper bound of $\log(N_O)$ on $K(x|f,n)$. Taken together, these arguments suggest the following range:

$$0 \leq K(x|f,n) \leq \log_2(N_O) + \mathcal{O}(1) \tag{22}$$

our upper bound equation (12) would then satisfy:

$$\frac{1}{N_O} \leq 2^{-K(x|f,n)+\mathcal{O}(1)} \leq 1. \tag{23}$$

The upper bound on our bound is in a sense trivial, as the probability of an output cannot be greater than 1. The lower bound is just the average value of $P(x)$ given that there are N_O outputs. Just to be clear, the lower bound above is not a lower bound for the probability $P(x)$ of an output: it is the expected *lowest* value for the *upper* bound $P(x) \leq 2^{-K(x|f,n)+\mathcal{O}(1)}$, which is a decreasing function of $K(x|f,n)$. In other words, the upper bound on $P(x)$ – which for any given $K(x|f,n)$ gives the upper bound on the probability of outputs with that value of $K(x|f,n)$ – can be expected to roughly vary between these two extremes as a function of $K(x|f,n)$, The actual probabilities $P(x)$, on the other hand, can be closer or further from the bound. For example, the bound above tells us that $P(x)$ for the most complex outputs will be less than $1/N_O$. This upper bound does not preclude there being that many x in this set for which $P(x) \ll 1/N_O$, in fact for highly biased distributions we might expect this to be the case for the high complexity outputs. If there is bias in the distribution there are likely many outputs within the whole set $x \in O$ that have $P(x) < 1/N_O$, since $\sum_{o=1}^{N_O} P(x_0) = 1$.

SUPPLEMENTARY NOTE 6

Sampling inputs produces outputs close to the bound

The upper bound on the probability to obtain a certain output on its own this does not say much about how close we expect an actual $P(x)$ to be to this bound. In this section, we derive a lower bound for $P(x)$ when x is *produced from a random input* (See also discussions in refs 3 and 1).

Consider a computable function $f(p) = x$, where p is some input program producing output x. Let $p \in \{0,1\}^n$, so that all inputs have length n. Define the set $A(x)$ to be the pre-image of x, i.e. the set of all the inputs that map to x, so that

$$P(x) = \frac{|A(x)|}{2^n} \tag{24}$$

We can describe any arbitrary input p using the following procedure: Assuming f and n are given, first enumerate all 2^n inputs and map them to outputs using f. Then describe the output $x = f(p)$ using $K(x|f,n)$ bits, and finally, describe the index of the specific input p within the set $A(x)$ using at most $\log_2(|A(x)|)$ bits. For example, if the set has 1024 elements, then with $\log_2(1024) = 10$ bits, we can describe any index $i = 1, 2, 3, \ldots, 1024$. In other words, this procedure basically means identifying each input by first finding the x it maps to, and then finding its label within $A(x)$.

Following this procedure allows us to write the following bound for the complexity of the input p:

$$K(p|n) \leq K(x|f,n) + \log_2(|A(x)|) + \mathcal{O}(1) \tag{25}$$

If we choose a random input p, then with high probability (i.e. for most inputs) we will have $K(p|n) = n + \mathcal{O}(1)$, and the inequality becomes

$$n \leq K(x|f,n) + \log_2(|A(x)|) + \mathcal{O}(1) \tag{26}$$

Rearranging yields

$$2^{-K(x|f,n)-\mathcal{O}(1)} \leq |A(x)|/2^n = P(x) \tag{27}$$

Combining this lower bound together with the upper bound of equation (12) shows that for a randomly chosen input we have

$$2^{-K(x|f,n)-\mathcal{O}(1)} \leq P(x) \leq 2^{-K(x|f,n)+\mathcal{O}(1)} \tag{28}$$

with high probability. Note that both sides of the bound depend on uncontrolled $\mathcal{O}(1)$ terms. They arise from similar procedures, but are different, and we have put a minus sign on the left side of the equation to make this clear. Nevertheless, the overall argument suggests that for x generated by randomly chosen inputs, the probability $P(x)$ should not be too far off from our upper bound. Indeed, the direct calculations of the maps in the main paper show that most of the probability mass is found not too far (on a log scale) from the upper bound.

Another way to analyse the proximity of $P(x)$ to the upper bound is to define the function

$$q(x) = \frac{2^{-K(x|f,n)+\mathcal{O}(1)}}{P(x)} \tag{29}$$

which measures the ratio of the upper bound of equation (12) to the probability $P(x)$ that an output x is generated by random sampling of inputs. $q(x)$ measures the relative overestimate of the probability of output x when we approximate it by the upper bound, so we expect that in general $q(x) \geq 1$.

The expected value of $q(x)$ summed over all inputs, which we call $\mathcal{E}_I$, can be written as a sum over all outputs, where every output is weighed as $P(x)$:

$$\mathcal{E}_I = \frac{1}{N_I}\sum_{i=1}^{N_I} q(x(p_i)) = \sum_{i=1}^{N_O} P(x_i)q(x_i) \tag{30}$$

$$= \sum_{i=1}^{N_O} 2^{-K(x_i|f,n)+\mathcal{O}(1)} \tag{31}$$

For a computable map $\sum_{x\in O} P(x) = 1$. Because $K(x|f,n)$ is a prefix code, $\sum_{x\in O} 2^{-K(x|f,n)} \leq 1$, but since $q(x) \geq 1$ we know that $\mathcal{E}_I = \sum_{x\in O} 2^{-K(x|f,n)+\mathcal{O}(1)} \geq 1$ due to the $\mathcal{O}(1)$ terms.

More generally, since $\mathcal{E}_I$ is finite for a computable map, and $q(x) \geq 0$, we can use Markov's inequality[18], which implies, for $q(x)$ generated by random inputs, that [3]:

$$\sum_{i=1}^{N_O} \{P(x_i) : q(x_i) > \mathcal{E}_I r\} < \frac{1}{r} \tag{32}$$

for $r > 0$. From this it immediately follows that:

$$\sum_{i=1}^{N_O} \left\{P(x_i) : \frac{2^{-K(x_i|f,n)+\mathcal{O}(1)}}{\mathcal{E}_I r} \leq P(x_i)\right\} \geq 1 - \frac{1}{r} \tag{33}$$

In other words, on uniform random sampling of inputs, the lower bound in

$$\frac{2^{-K(x|f,n)+\mathcal{O}(1)}}{\mathcal{E}_I r} \leq P(x) \leq 2^{-K(x|f,n)+\mathcal{O}(1)} \tag{34}$$

holds with a probability of at least $1 - \frac{1}{r}$, while the upper bound, given by equation (12), always holds. Since we find that $P(x)$ typically varies by many orders of magnitude, we will consider the bound to be tight (on a log scale) if it is within one or two orders of magnitude of the true $P(x)$. Another way of thinking about this lower bound is to observe that $\mathcal{E}_I$ is the input averaged ratio of the bound to the true $P(x)$. We measured $\mathcal{E}_I$ explicitly for the maps in the main text compared to our approximate upper bound and find that typically $\log_{10}\mathcal{E}_I \approx 1$ or 2, so that, using our definition above, the bound is relatively tight when random inputs are chosen for the maps described in the main text.

We emphasise that the fact that random sampling of inputs generates $P(x)$ close to the upper bound does not mean that most outputs x lie close to the bound. The average probability over all N_O outputs is still $< P(x) >= 1/N_O$, while we find for our upper bound $2^{-K(x|f,n)+\mathcal{O}(1)} \gg 1/N_O$ for low complexity outputs, and even for the largest $K(x|f,n)$ the upper bound $\geq 1/N_O$. So if the bound is tight for the outputs generated by random sampling of inputs, then these outputs typically have $P(x) \gg 1/N_O$. To compensate, there must be many outputs with $P(x) < 1/N_O$. Thus while sampling random inputs generates outputs that are relatively close to the bound, sampling random outputs uniformly should give many $P(x)$ that are typically well below the bound. Indeed, for the maps we describe in this paper this behaviour is observed.

SUPPLEMENTARY NOTE 7

Approximations to $K(x)$

Kolmogorov complexity $K(x)$ is formally uncomputable[1]. At best, it can be approximated from above. At first sight these properties might seem to make it impractical to use. Nevertheless, there is a significant literature that uses various approximations to $K(x)$ which have been found to work remarkably well in various applications, see e.g. references[19–31]

We follow this same approach here, and in particular use an influential complexity measure for digital strings (or sequences) that was introduced in 1976 by Lempel and Ziv[32]. Their algorithm forms the foundation for many popular

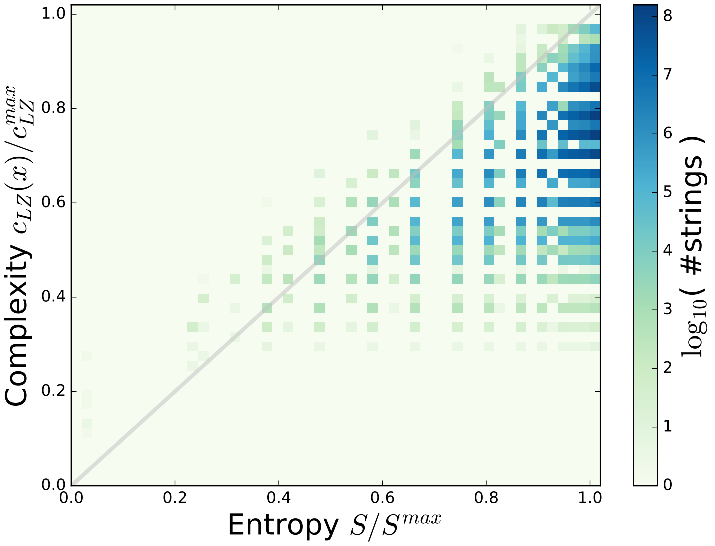

Supplementary Figure 1. Heatmap for the complexity $C_{LZ}(x)$ versus entropy $S(x)$ for binary strings of length $n = 30$. Both measures are normalised by their maximum value. Complexity is bounded by entropy, in the sense that a binary string with mostly zeros (and therefore low entropy) cannot be complex. Conversely, a string such as 010101...01 has maximum entropy, since it is made of an equal number of 0s and 1s (thus $S/S^{max} = 1$), while still being very simple ($C_{LZ}(x)/C_{LZ}^{max} \approx 0.273$).

compression algorithms. The essence of the Lempel-Ziv algorithm is to read through a string (of any finite alphabet size) from left to right and create a dictionary of new sub-patterns as they appear in the string. A string with many different sub-patterns would then yield a large dictionary, and hence be assigned a high complexity. Conversely, a string of little variation that is essentially built up of repeated sub-patterns would yield a small dictionary, and hence would be assigned a low complexity. If the number of words (distinct patterns) in the dictionary is $N_w(x)$ then Lempel and Ziv showed subsequently[30,33] that for an ergodic source and in the limit of long sequences that

$$\lim_{n\to\infty} \frac{N_w(x)\log_2(n)}{n} = \frac{K(x)}{n} = h(x) \tag{35}$$

for nearly all sequences, where $n = l(x)$ is the length of the binary strings, and h is the standard Shannon entropy *rate*. This complexity function has thus been a popular choice for approximating Kolmogorov complexity in the literature. In particular, it is thought to work better than other lossless compressions based measures for shorter strings[26,34].

We use the following approximate complexity measure based on the 1976 Lempel Ziv algorithm[32]:

$$C_{LZ}(x) = \begin{cases} \log_2(n), & x = 0^n \text{ or } 1^n \\ \log_2(n)[N_w(x_1...x_n) + N_w(x_n...x_1)]/2, & \text{otherwise} \end{cases} \tag{36}$$

The reason for distinguishing 0^n and 1^n is merely an artefact of $N_w(x)$ which assigns complexity $K = 1$ to the string 0 or 1, but complexity 2 to 0^n or 1^n for $n \geq 2$, whereas the Kolmogorov complexity of such a trivial string actually scales as $\log_2(n)$, as one only needs to encode n. In this way we ensure that our $C_{LZ}(x)$ measure not only gives the correct behaviour for complex strings in the $\lim_{n\to\infty}$, as shown in equation (35), but also the correct behaviour for the simplest strings. In addition to the $\log_2(n)$ correction, taking the mean of the complexity of the forward and reversed strings makes the measure more fine-grained, since it allows more values for the complexity of a string. Note that $C_{LZ}(x)$ can also be used for strings of larger alphabet sizes than just 0/1 binary alphabets.

It is instructive to compare our measure $\tilde{K}(x) = C_{LZ}(x)$ to the simple binary entropy, defined as $S(x) = p\log p + (1-p)\log(1-p)$, where p is the fraction of 1s (or 0s) in the string x. While low entropy strings typically have low $\tilde{K}(x)$, the converse is not always true. Strings that are algorithmically simple can still have high entropies, as we illustrate in Supplementary Figure 1. Nevertheless, as is well known in the literature (see also equation (35)), in the limit of long strings, the mean Kolmogorov complexity per length tends to the entropy rate.

In Supplementary Figure 2a we plot the probability distribution of complexities for a wide range of lengths n. For lengths $n \leq 30$ we performed complete enumerations, and for longer lengths we performed sampling with 1×10^9 samples for each length. We estimated the mean complexity, the modal complexity, and the standard deviation as a

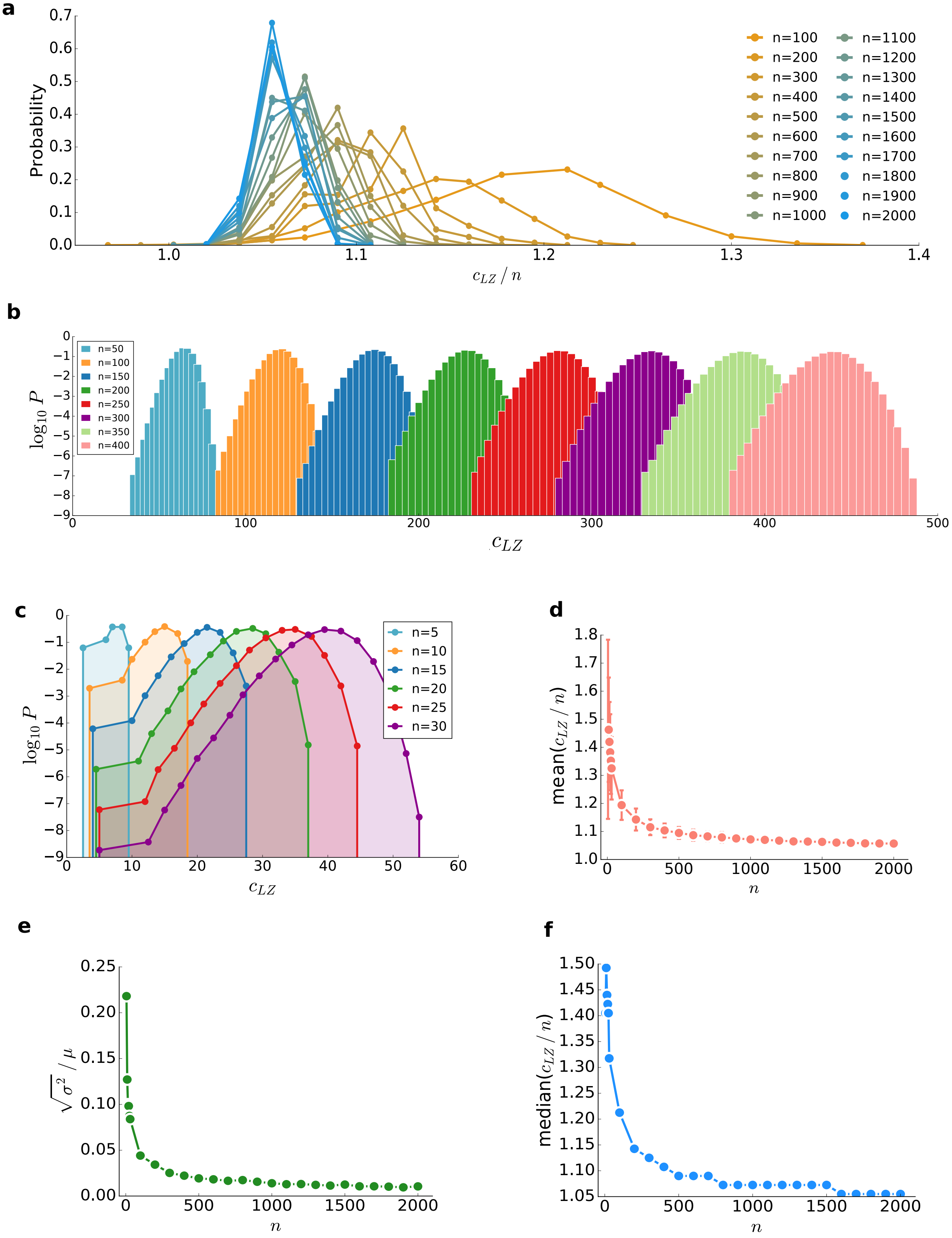

Supplementary Figure 2. **Distribution of complexity values calculated with $C_{LZ}(x)$ for strings of different length n.** All strings of length $n = \{5, 10, 15, 20, 25, 30\}$ were enumerated, and for $n > 30$ samples of 10^9 strings were taken. **(a)** Distribution of $C_{LZ}(x)/n$, for $n = 100$ to 2000. **(b)** Distribution of $C_{LZ}(x)$, for $n = 50$ to 400. **(c)** Distribution of $C_{LZ}(x)$, for $n = 5$ to 30. **(d)** Mean of $(C_{LZ}(x)/n)$, for $n = 5$ to 400. Error bars represent one standard deviation. **(e)** Standard deviation σ over the mean μ of $C_{LZ}(x)$, for $n = 5$ to 400. **(f)** Median of $(C_{LZ}(x)/n)$, for $n = 5$ to 400. Note that as n grows, the mean and median C_{LZ}/n approach 1, and the standard deviation σ over the mean μ drops: In other words, the distribution becomes more peaked. Lines connecting data points were added to guide the eye.

function of the length n. Also, we show in Supplementary Figure 2e that as the length of the strings get longer, the distributions get relatively narrower. The mean is expected to approach $n \to \infty$ limit $< C_{LZ} > \approx n$[30,33]. The main thing to note is that for a given n, strings with complexity well below the mean are rare, and progressively more rare for lower complexities.

Any estimator of Kolmogorov complexity will have weaknesses. And there are some subtleties that should be kept in mind when interpreting $C_{LZ}(x)$. Firstly, rather than most strings having the maximum complexity, we find that the majority of strings are close to the mode which is in turn very near the mean complexity (See Supplementary Figure 2c,d,e). For Lempel-Ziv it is known that strings with maximal complexity are somewhat anomalous because they can be created by an algorithmic process[35], which is an artefact of the Lempel-Ziv algorithm itself, and so they are in fact not the most complex in a Kolmogorov sense. However, as can be seen in Supplementary Figure 2 these highest complexity strings remain rare, and so for our purposes they do not play a big role.

We also applied two alternate complexity measures to $C_{LZ}(x)$. Firstly, in Supplementary Figure 3 we apply the `Compress` function in *Mathematica* to RNA secondary structures of $n = 55$ and $n = 80$ bases. As can be seen, the values of the complexity approximation $\tilde{K}(x)$ are different, so that the values of a and b, estimated using the methods described in Supplementary Note 8, are different, as expected. Nevertheless, the same basic simplicity bias phenomenology obtains, as we would predict. Note that `Compress` is similar to `zlib` compression, which is based on another of Lempel and Ziv's famous compression algorithms, often called `LZ77`[36].

Given that most lossless compression algorithms are influenced by the ideas of Lempel and Ziv, it is not straightforward to find measures that are truly different in origin. Recently, however, a fundamentally different way of estimating the Kolmogorov complexity of strings has been derived in an important series of papers[37–39] that apply the full AIT coding theorem by sampling over many Turing machines. In principle this coding theorem method (CTM) is very powerful, and in particular can go well beyond lossless compression techniques, which are fundamentally sophisticated entropy measures. However, CTM is limited to very short strings ($\lesssim 12$ bits). To calculate the complexity for longer strings, one can use the Block Decomposition Method (BDM) which, as the name suggests, breaks such strings into smaller blocks, whose complexity can be approximated by the value taken from the CTM[39,40]. As shown in Supplementary Figure 3, we again obtain the basic simplicity bias phenomenon. In this BDM case we simply fit to both a and b, rather than using the methods described in Supplementary Note 8. The reason is that some of the simplifying assumptions used in Supplementary Note 8 do not work for the BDM method. For example, we assume that a can be approximated by assuming b to be zero, but that does not work here, most likely because there is a larger additive constant to the BDM complexity than to the $C_{LZ}(x)$. However, as argued in Supplementary Note 8, additive and multiplicative changes can all be absorbed into a and b. In principle, once one has fit a and b for a given complexity measure, which only needs a small number of outputs, then equation (37) can be used for other outputs of the same map.

Finally, the coding theorem results which we invoke apply to the prefix-free version of Kolmogorov complexity, denoted $K(x)$, as opposed to the plain Kolmogorov complexity, denoted $C(x)$, see 1. However, while these two measures have important theoretical differences, they are asymptotically equal by equation (4), and so quantitatively close. Since we approximate $K(x)$ anyway, we ignore the subtle distinction between these measures in our approximations. Some of the differences may be absorbed into our parameters a and b, discussed in the next section.

SUPPLEMENTARY NOTE 8

Predicting a and b for computable maps

A key result in the main text is equation (3), which approximates the upper bound (12) for limited complexity maps as

$$P(x) \leq 2^{-a\tilde{K}(x)-b} \tag{37}$$

In this section, we describe how to make predictions for the values of a and b by using minimal information about the maps.

Estimating a

To derive an approximation for the slope a we start with equation (23) which provides lower and upper bounds on the probability bound. If we assume that the minimum upper bound is reached by the most complex objects, i.e. outputs such that our approximation to the Kolmogorov complexity, $\tilde{K}(x)$, takes the value $\max_{x\in O} \tilde{K}(x)$, and

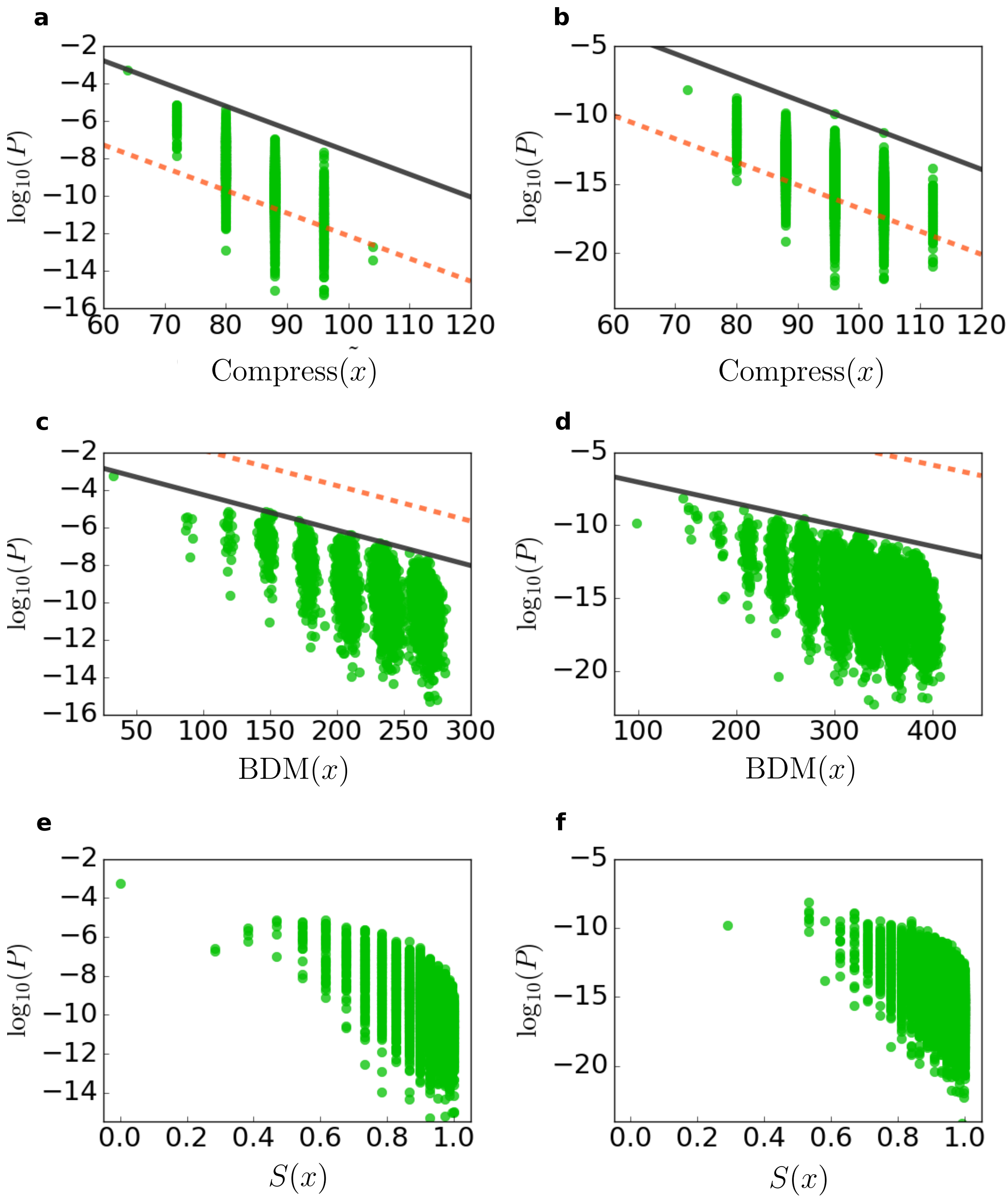

Supplementary Figure 3. **Simplicity bias predicted by other approximations to Kolmogorov complexity**. We use, *Mathematica*'s `Compress` function, the block decomposition method (BDM) and the simple entropy $S(x)$ of the dot-bracket notation, for $n = 55$ and $n = 80$ RNA secondary structures. In order, the plots show **(a)** `Compress` for $n = 55$ RNA, **(b)** `Compress` for $n = 80$ RNA, **(c)** BDM for $n = 55$ RNA, **(d)** BDM for $n = 80$ RNA, **(e)** Entropy $S(x)$ for $n = 55$ RNA, **(f)** Entropy $S(x)$ for $n = 80$ RNA. The solid lines denote our estimated upper bound, the dashed lines are the upper bound with $b = 0$. For the Compress and BDM method, we observe a similar simplicity bias phenomenology to what was observed in Figure 1a of the main paper, where C_{LZ} was used. For the entropy measure, there is also a decay of the probability with increasing complexity, but the behaviour of the upper part of the curves are significantly less linear than for Compress, BDM and $C_{LZ}(x)$.

further that b can be ignored for this derivation which we motivate below, then a has a simple approximation, shown as equation (4) in the main text:

$$a \approx \frac{\log_2(N_O)}{\max\limits_{x \in O}(\tilde{K}(x))} \tag{38}$$

Using this equation we can either find the gradient a from knowing N_O, or find N_O from knowing a. Alternatively, if we have a way of estimating $\max(\tilde{K}(x))$, as well as the gradient a, then we can infer N_O directly. Since N_O is very hard to estimate for large maps where exhaustive enumerations are not possible, this method may be a way to get a quick estimate of N_O based on some limited sampling.

Estimating b

As a first approximation, we note that if a map is strongly biased towards simple outputs, then we expect the largest $P(x)$ for outputs x with complexity for $\tilde{K}(x) = \min\limits_{x}(\tilde{K}(x))$ to be at most within one or two orders of magnitude of 1. That suggests that b is generally small, and as a zeroeth order approximation we assume that $b \approx 0$.

Alternatively, if $P(x)$ is known for some output x, then assuming knowledge of a and $\tilde{K}(x)$, and assuming

$$P(x) \approx 2^{-a\tilde{K}(x)-b} \tag{39}$$

then b can be inferred by rearranging this equation. Clearly this method would work just as well for finding a, if one knew b and $\tilde{K}(x)$. It seems plausible that the most likely $P(x)$ to be close to the upper bound is the largest $P(x)$ for the set of x with modal $K(x)$, i.e. the $K(x)$ that is most likely to be generated by sampling random inputs. We typically use this value of $P(x)$ to fix b. One drawback with this method is that it relies on the assumption of the approximate equality equation (39); hence if for the chosen output x, the upper bound was only a poor approximation, then the corresponding estimation of b would be equally poor.

With the methods above, reasonable approximations to a and b can generally be estimated with a limited amount of sampling of random inputs, as we demonstrate in the main text. As long as there are ways to estimate $\max(\tilde{K}(x))$, and N_O, then the only real fitting parameter is b, which to first order can simply be set to zero. Of course some simplifying assumptions have been used here. Not all maps may obey them, but we can always simply fix a and b with a few values of $P(x)$ and $\tilde{K}(x)$. It remains the case that only a small amount of information is needed to fix the bound.

Finally, we note that the values of a and b depend on the chosen approximate measure of complexity. In this paper we use $K(x) \approx \tilde{K}(x) = C_{LZ}(x)$. If we were to choose a different complexity say $\tilde{K}_{\alpha,\beta} = \alpha C_{LZ}(x) + \beta$, then the phenomenology would be the same, but with new constants $a_{\alpha,\beta} = a/\alpha$ and $b_{\alpha,\beta} = b - a\beta/\alpha$. In other words, multiplicative and additive constants are simply absorbed into the parameters. Such robustness is a useful property.

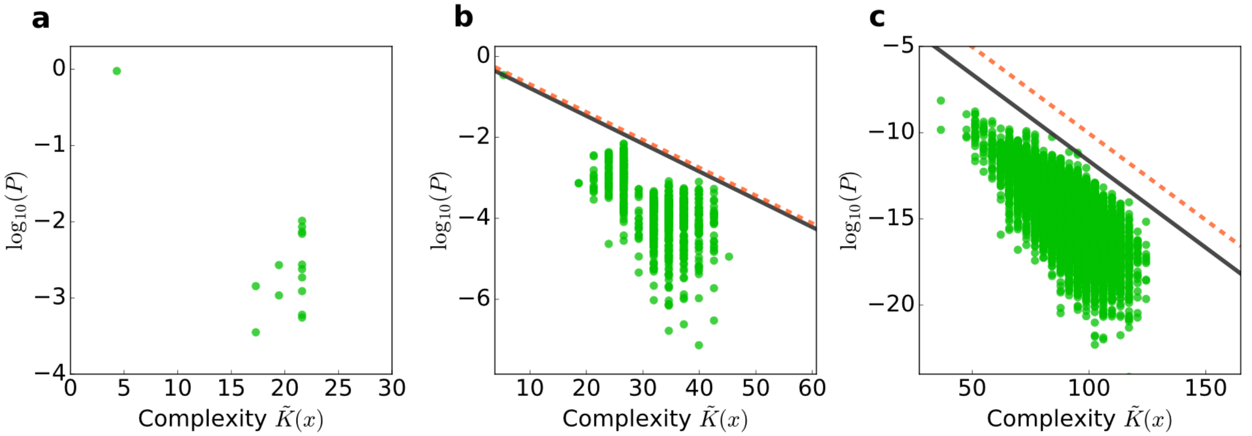

Supplementary Figure 4. **Probability $P(x)$ vs. increasing complexity for different sized systems.** **(a)** RNA $n = 10$ shows essentially no simplicity bias, although the trivial unbonded and simplest structure does have the largest probability. **(b)** RNA $n = 20$ shows simplicity bias, despite the noise. For the upper bound, $a = 0.23$, $b = 1.08$; **(c)** RNA $n = 80$ shows clear simplicity bias, as does $n = 55$ in the Main text. For the upper bound, $a = 0.33$, $b = 6.39$.

SUPPLEMENTARY NOTE 9

Simplicity bias and system size

In the main text we argue that one needs $N_O \gg 1$ to avoid finite size effects when measuring the simplicity bias of an input-output map. Here we illustrate this point, showing finite-size effects in the RNA map. Supplementary Figures 4a, b and c respectively show $\log P(x)$ vs. $K(x)$ plots for $n = 10, 20$ and 80 RNA sequences, supplementing the plot for $n = 55$ map in the main text. The plots for the shortest sequences show behaviour that deviates from the upper bound simplicity bias prediction: Supplementary Figure 4a with $n = 10$ RNA shows no simplicity bias, except for the trivial structure of no bonds which is simplest and highest in probability, Supplementary Figure 4b with $n = 20$ shows simplicity bias, but with some noise. In contrast, Supplementary Figures 4c for $n = 80$ and Figure 1(a) from the main text ($n = 55$) show pronounced simplicity bias for a range of values for $\tilde{K}(x)$. Thus as N_O grows, simplicity bias becomes clearer.

For the shorter systems, full enumerations are possible, but since the space grows as $N_I = 4^n$, this is not possible for longer systems. For example, for $n = 55$, there are $4^{55} \approx 10^{33}$ different structures and an estimated 10^{13} different secondary structure outputs[41]. As it can be seen in Supplementary Figure 5, we only sample a small fraction of the total number of outputs, and these are typically those with higher $P(x)$.

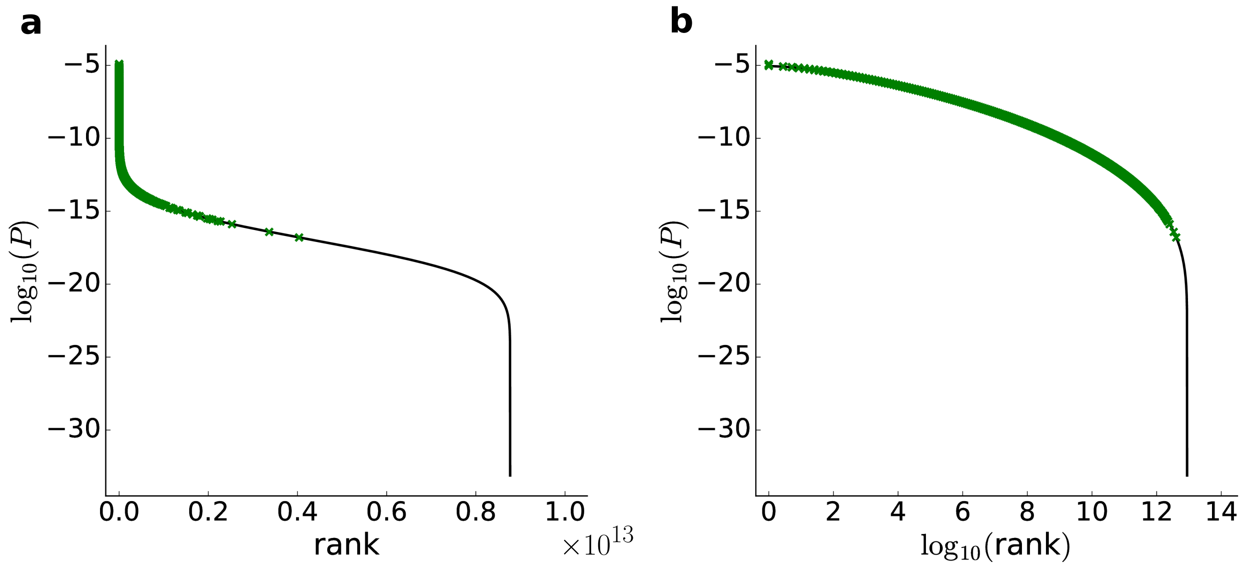

Supplementary Figure 5. Probability $P(x)$ that a phenotype is obtained by random sampling over inputs versus its rank for RNA secondary structures, in **(a)** linear and **(b)** logarithmic scale. In black, we show the analytic approximation for the probability distribution derived in ref. 41. In green, the probability of sampled RNA structures of length $n = 55$ shown in Figure 1a in the main text. Note that structures of low probability are not found by sampling.

SUPPLEMENTARY NOTE 10

Predicting which of two outputs has higher probability

The arguments above suggest that if outputs x and y are generated from random sampling of inputs, so that the outputs are expected to be close to their upper bounds, and if $\tilde{K}(x) < \tilde{K}(y)$ holds, $P(x) > P(y)$ should also hold in most cases. We tested this claim for all input-output maps in the Figure 1 in the main paper by sampling 10^4 pairs of outputs (x, y), and counting a prediction as 'correct' if the claim holds or if $\tilde{K}(x) = \tilde{K}(y)$ and $P(x)$ is within a factor of 10 of $P(y)$. Naturally, the null hypothesis would be no bias towards simple outputs therefore we should obtain $P(x) > P(y)$ for 50% of the samples. We performed output-sampling, i.e. weighing every output equally, and input-sampling, i.e. weighing every output by its probability. Here is the percentage of correct results for each input-output map from Figure 1 in the main, including the complex matrix map, for which this method does not

work, since it does not show simplicity bias:

- RNA: input-sampled 99%, output-sampled 78%
- Circadian rhythm: input-sampled 81%, output-sampled 71%
- Ornstein-Uhlenbeck financial model: input-sampled 92%, output-sampled 75%
- L-systems: input-sampled 87%, output-sampled 68%
- Simple matrix map: input-sampled 89%, output-sampled 71%
- Complex matrix map: input-sampled 49%, output-sampled 52%

For the simple matrix map, we ignored the highest probability output (a trivial vector) since this takes up the majority of the space and so skews the $P(x)$ v.s. $P(y)$ results. But overall, we see that just using $K(x)$ and $K(y)$ can give a good first guess of whether $P(x)$ is larger of smaller than $P(y)$. Of course, the larger the difference $K(x)-K(y)$, the more confidence we can have in the difference between $P(x)$ and $P(y)$.

SUPPLEMENTARY NOTE 11

Low complexity, low probability outputs have lower complexity inputs

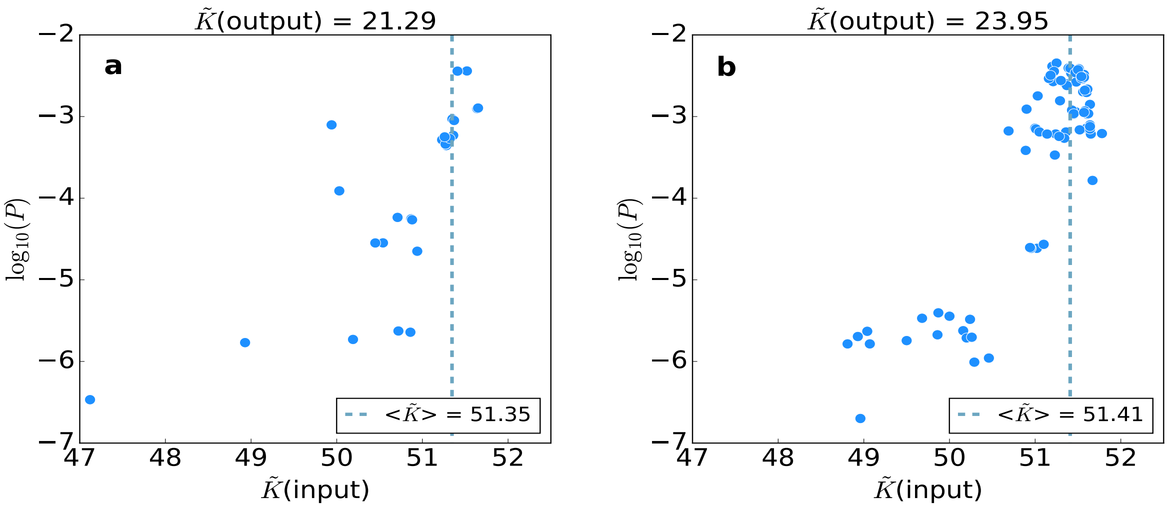

Supplementary Figure 6. The probability $P(x)$ to obtain a given RNA structure x upon random sampling of inputs as a function of the mean complexity of the input sequences that produce x for $n = 20$ RNA, shown for **(a)** $\tilde{K}(x) = 21.29$ and **(b)** $\tilde{K}(x) = 23.95$. These are among the lowest complexity values, as seen in Supplementary Figure 4b. As predicted by equation (25), secondary structure outputs with a lower $P(x)$ (further from the bound) have a lower than average sequence complexity.

There is another implication from the upper bound on the complexity of an input, described in equation (25): if both $K(x|f,n)$ and $P(x) = |A(x)|/2^n$ are small, then this equation will be violated unless the l.h.s. is also small. In other words, this argument also predicts that low complexity outputs with low probability should be generated by inputs for which $K(p|n) < n$, i.e. inputs that are simpler than what we obtain by random sampling inputs. Small $K(x|f,n)$ suggests a larger upper bound on $P(x)$, so the outputs with simpler input sets are those for which $P(x)$ is far from the upper bound of equation (12).

To illustrate this effect we take the RNA map as an example. We measure the complexity K_I of an RNA input string by replacing each of the 4 nucleotide letters with `00`, `01`, `10`, or `11`, and then measure the complexity of the corresponding binary sequence. We then sampled 50 million RNA sequences with $n = 20$ nucleotides, and measured the mean complexity of the inputs associated to each output. For randomly chosen $n = 10$ RNA input strings we

find an average complexity of $K_I = 51.2$. However, as can be seen in Supplementary Figure 6, low $K(x)$ low $P(x)$ outputs, i.e. ones are far from the upper bound of Equation (12), have an average sequence complexity $K_I < 51.2$, whereas those outputs that are closer to the upper bound, have the expected average complexity close $K_I = 51.2$, the average for randomly chosen RNA $n = 20$ input strings. Since, as discussed above, low complexity strings are rare, the number of inputs mapping to low $K(x)$ low $P(x)$ outputs must be a small fraction of all input strings. This in turn implies that random inputs (which are overwhelmingly complex) must mainly map to outputs where $P(x)$ is not too far from the bound.

The validation of this non-trivial prediction from equation (25) helps justify our arguments above for the other conclusion that follow from this equation, namely that randomly sampled inputs are likely to generate outputs x with probabilities $P(x)$ nearer the upper bound of Equation. (12).

SUPPLEMENTARY NOTE 12

Simplicity bias in other input-output maps

In this section we provide some more examples of input-output maps, and elaborate further on some maps discussed in the main text.

The circadian rhythm model

In the main paper we study a well known model by Vilar et al.[42] for the circadian rhythm of eukaryotes as an input-output map. The model consists of a set of nine equations:

$$
\begin{aligned}
dD_A/dt &= \theta_A D'_A - \gamma_A D_A A \\
dD_R/dt &= \theta_R D'_R - \gamma_R D_R A \\
dD'_A/dt &= \gamma_A D_A A - \theta_A D'_A \\
dD'_R/dt &= \gamma_R D_R A - \theta_R D'_R \\
dM_A/dt &= \alpha'_A D'_A + \alpha_A D_A - \delta_{M_A} M_A \\
dA/dt &= \beta_A M_A + \theta_A D'_A + \theta_R D'_R \\
&\quad - A(\gamma_A D_A + \gamma_R D_R + \gamma_C R + \delta A) \\
dM_R/dt &= \alpha'_R D'_R + \alpha_R D_R - \delta_{M_R} M_R \\
dR/dt &= \beta_R M_R - \gamma_C AR + \delta_A C - \delta_R R \\
dC/dt &= \gamma_C AR - \delta_A C
\end{aligned} \tag{40}
$$

Since we are mainly using this model to illustrate a generic ODE map, we will not give a complete description of what all the parameters mean. For a full account we point to the original paper[42]. Very briefly, the model above aims to study the ability of circadian clocks to maintain a constant period even in noisy conditions, and describes the interaction of two genes that regulate the expression of a pair of proteins, the activator A and the repressor R. This repressor works by sequestering the activator, forming the inactivated complex C, whose concentration over time is the variable we chose to use as output, taking its rate of formation (i.e, its slope) at discrete time steps to produce our binary string. Since this variable is placed at the bottom of the regulatory cascade, it is a natural choice for the output. Supplementary Figure 7 represents the output of the ODE model, showing the concentration of the nine molecules over time, for the set or parameters given in the original paper[42]: D_A and D_R start at 1 molecule, meaning one single copy of the activator and repressor genes, and other variables start at zero, with $\alpha_A = 50$ h^{-1}, $\alpha'_A = 500$ h^{-1}, $\alpha_R = 0.01$ h^{-1}, $\alpha'_R = 50$ h^{-1}, $\beta_A = 50$ h^{-1}, $\beta_R = 5$ h^{-1}, $\delta_{M_A} = 10$ h^{-1}, $\delta_{M_R} = 0.5$ h^{-1}, $\delta_A = 1$ h^{-1}, $\delta_R = 0.2$ h^{-1}, $\gamma_A = 1$ molecule^{-1} h^{-1}, $\gamma_R = 1$ molecule$^{-1} h^{-1}$, $\gamma_C = 2$ molecule^{-1} h^{-1}, $\theta_A = 50$ h^{-1}, and $\theta_R = 100$ h^{-1}.

Since in this input-output map the inputs are given as continuous parameters, it is less clear how to sample inputs than in cases such as RNA where the inputs are discrete strings. Here, instead, every input parameter corresponds to a biological constant or rate, and the realistic ranges for such parameters are often unknown. Moreover, the value of each parameter – allosteric constants, affinity rates – is also the product of a series of other very complex input-output maps, transducing information from DNA sequences into amino acid sequence and eventually into a parameter in this ODE model. To make progress, and since we are simply treating this as a model map, we set all 15 parameters to

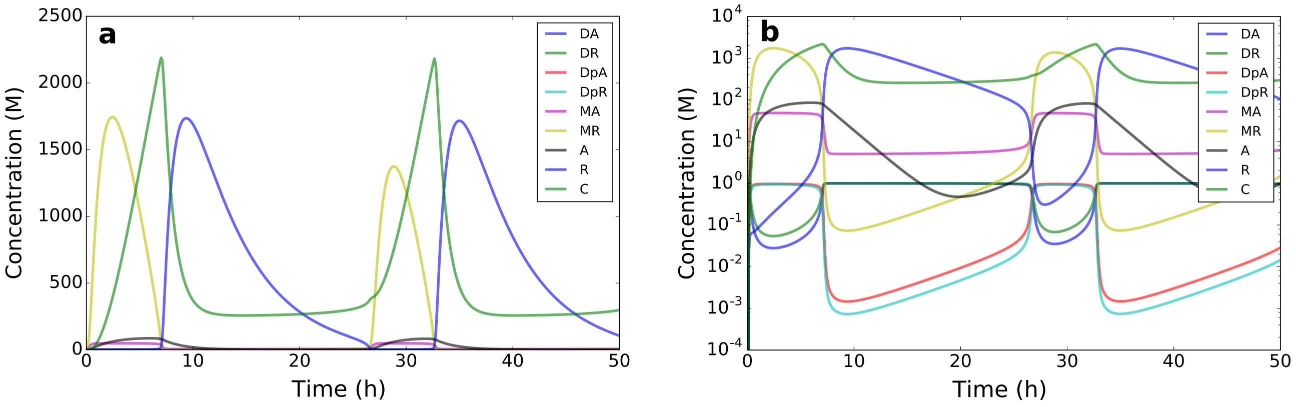

Supplementary Figure 7. The output of the ODE model described in equation (41), showing the concentration of 9 molecules from equation (40) over time, in linear scale **(a)** and logscale **(b)**. The initial conditions and parameters are the same as in the original paper[42].

their original values, multiplied by a random factor in $\{0.25, 0.50, \ldots, 1.75, 2.00\}$ chosen with uniform probability. In this way we effectively have a discrete set of input parameters.

Since the outputs depend continuously on the input variables, in principle every input would produce a unique output. Nevertheless, intuitively, many outputs can be very similar to one another. To capture this, we coarse-grain the outputs by discretising them into binary strings using the "up-down" method[43,44]. We take the output curve $y(t)$ calculated in an interval $t \in [0, T]$, calculate its slope dy/dt at intervals of $t = \delta t, 2\delta t, 3\delta t, \ldots$, and print the sign of dy/dt in every interval: for $j = 1, \ldots, T/\delta t$, if $dy/dt \geq 0$ (or < 0) at $t = j\delta t$, the j-th bit of the output string gets assigned a 1 (or a 0). The resulting string represents the oscillations of $y(t)$: curves with more oscillations will produce more complex strings, while curves with fewer oscillations will produce strings with longer repeated sequences of 0s and 1s.

Alternative sampling of inputs

To check that our discretisation method does not affect the main effects we observe, we also take the same range of parameters, but now randomly sample uniformly on the whole range for each one, e.g. not just or discrete values. In Supplementary Figure 8a we used this method to randomly sample 10^6 inputs and used the same method as above to calculate the complexities of the outputs. As can be seen, the same simplicity bias behaviour obtains as can be seen in Figure 1b of the main paper. The values of a and b for the two sampling methods agree more or less within the uncertainty of our methods for obtaining them.

Discretising initial conditions

Since the behaviour described by the circadian rhythm model should depend on the initial conditions of the ODE system, it is important to test whether the simplicity bias we observe also depends on that. We took a sample of 10^6 combinations of different values for parameters and initial conditions, produced their corresponding outputs and derived the upper bound coefficients $a = 0.32$ and $b = 1.39$ according to equation (38). The plot in Supplementary Figure 8b shows the expected simplicity bias phenomenology: there is a very biased distribution in the probability that correlates with complexity.

Alternative sample sizes

To generate Figure 1b for the ODE in the main paper, we took a sample of 10^6 inputs, which is less than total $8^{15} \approx 3 \times 10^{13}$ inputs for this coarse-grained ODE model. Consequently, any estimate of $P(x)$ for outputs for which $P(x) \lesssim 10^{-6}$ will be subject to large errors. Importantly, however, as can be seen in Supplementary Figure 9, even with as few as 10^3 samples, an upper bound can be identified which has a slope very close to the one found with much more sampling. We note that for the very small sample sizes, using the estimated N_O needed in equation (38) does

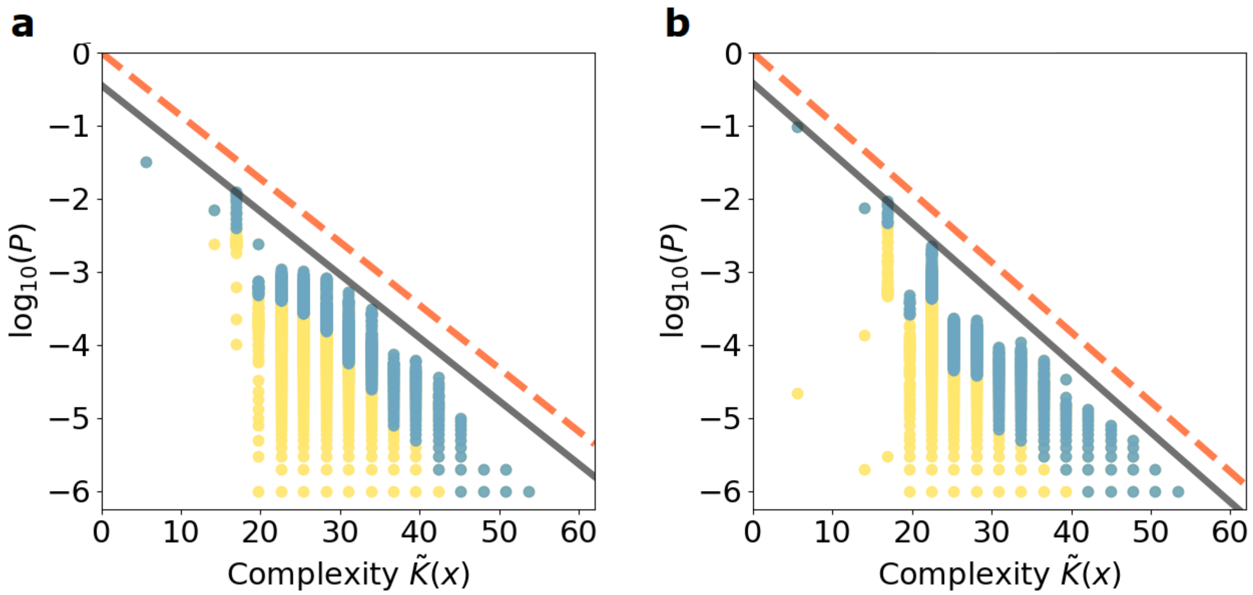

Supplementary Figure 8. **(a)** Probability versus complexity for the outputs of a circadian rhythm model with 10^6 inputs sampled uniformly from a continuous range, instead of sampled from discretised values over the same range. The upper bound coefficients $a = 0.29$ and $b = 1.48$ are derived according to equation (38) **(b)**Probability versus complexity for the outputs of a circadian rhythm model with 10^6 combinations of inputs and initial conditions, sampled from a discretised range. The upper bound coefficients $a = 0.32$ and $b = 1.39$ are derived according to equation (38). For both plots, outputs were discretised in 50 bins, and the results are very close to Figure 1b in the main text. For every value of $\tilde{K}$, the blue dots represent the outputs corresponding to 50% of the inputs that produce outputs with $\tilde{K}(x) = \tilde{K}$.

not work very well, but for the larger samples this works better and a consistent result is obtained. Nevertheless, we see that limited sampling is enough to identify the slope by fitting, and without needing equation (38). Hence the upper bound slope can be estimated without a full enumeration of the inputs, but with only partial sampling.

Cell cycle

Another input-output map we explore here is based on the well known the model of ref. 45, which describes part of the cell cycle for budding yeast. This well-established model is made of a large system of approximately 50 differential equations, where the values of more than 130 input parameters define the outputs, which are concentration-time curves for different chemicals. As an output we chose the concentration-time curve of the cell division cycle protein 6, or Cdc6. Our choice was motivated only by the observation that the Cdc6 curve varies sufficiently to yield many different outputs of varying complexity. To coarse-grain the outputs, we discretise the output curves to binary strings following the 'up-down' method[43,44] described above, yielding a binary string of length 40.

As inputs for this map also consist in real parameters of an ODE system, we take an approach akin to what we did for the circadian rhythm, and similar to what is done by the authors of this model in a robustness analysis[45]. We set all parameters to default values, and then sample by allowing each parameter to be scaled by a random factor $\sqrt{2}^{\zeta}$, where ζ is an integer $\{-4, , 4\}$, chosen with uniform probability. We then sampled 10^6 input parameter sets and produced the outputs shown in Supplementary Figure 10. As for the previous input-output maps, this one also shows clear simplicity bias.

Ornstein-Uhlenbeck financial model

In the main text we discuss an input-output map describing the pattern of price fluctuations modelled by the Ornstein-Uhlenbeck process. This process is described by the equation $dS_t = \theta(\mu - S_t)dt + \sigma dW_t$, where S_t is the price, μ and σ are parameters representing the historical average price and the market volatility respectively, θ is the noise dissipation rate, and W_t is a Brownian motion representing market noise. This Brownian motion is also the input of the map. These inputs then generate S_t, and the outputs x_t are defined also as sequences over n time steps,

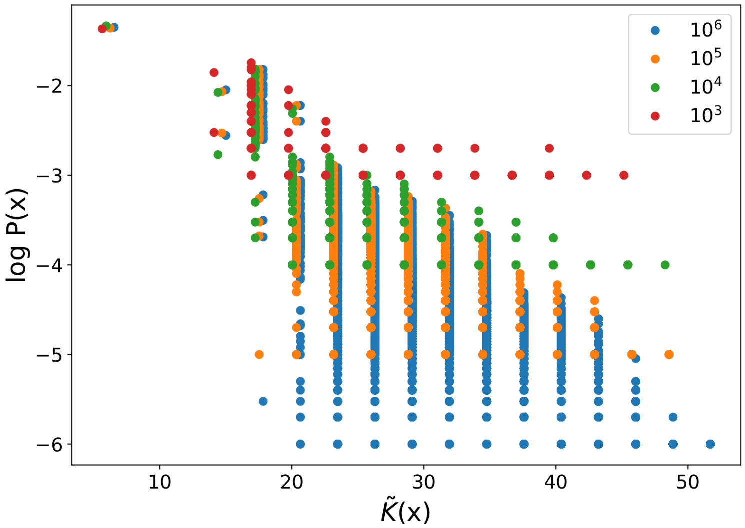

Supplementary Figure 9. Probability versus complexity for the outputs of a circadian rhythm model with 10^3 to 10^6 inputs, and outputs discretised in 50 bins. Note that for clarity, every sample size is displaced horizontally from the previous one by 0.3 for clarity. For sampling 10^n times the lowest possible probability is 10^{-n}, which explains the long $K(x)$ range at the minimum probability for each number of outputs. These are outputs that only appear once in the sample, and so the probability estimate is inaccurate. As the size of the input sample grows, the estimate of the probability of the rare outputs becomes more accurate. On the other hand, the $P(x)$ close to the upper bound do not change as much, and nor does the estimated values of a and b for the upper bound.

where $x_j = 0$ if $S_j \leq 0$, and $x_j = 1$ otherwise. The output sequence x can be interpreted as indicating whether S_t is above or below its historical average, and thus whether the trader would profit by selling or buying more of it.

In the main paper, we show that this input-output map shows simplicity bias phenomenology. In this section, we confirm that this bias towards simplicity we observe is not an artefact of our choice of parameters: Supplementary Figure 11 shows that different values of the parameters θ and σ still produce bias towards simple behaviour.

Note that the Brownian motion in this model could be coarse grained as a random walk of fixed step sizes, which in turn can be defined by a sequence of ± 1 steps. Both processes become equivalent when $\theta = 0$ in the Ornstein-Uhlenbeck process, causing the noise term σdW_t to dominate. From this perspective, we could also define the inputs in this model as the collection of all ± 1 sequences of some specified length, depending on the level or coarse graining. Because of that, this input-output map is also related to the random walk return map below, which describes a map from a one-dimensional random walk with fixed step size to a binary string output, where the j-th bit represents whether on step j the random walking particle is to the right ($s_j > 0$) or to the left ($s_j \leq 0$) of the origin $s_0 = 0$.

Random walk return map

In this section we study a simple one-dimensional random walk starting at $s_0 = 0$, followed by a series of uncorrelated steps of 1 or -1 as an input-output map.

Inputs are defined as follows: A walk of m steps will produce a position vector $s = (s_1, s_2, \ldots, s_m)$, where s_j represents the position of the random walker at time j. Since each sequence of steps is equally likely, and will produce a unique path, we consider this path as an input.

Outputs are defined as another sequence over m time steps, $x = (x_1, x_2, \ldots, x_m)$, where $x_j = 0$ if $s_j \leq 0$, and $x_j = 1$ otherwise. The changes from 0 to 1 (and vice-versa) in the output sequence thus correspond to times when the random walker returns to $s = 0$. We measure the complexity of these binary output strings in the usual way using $C_{LZ}(x)$.

One way of seeing this map is as a simplification of the Ornstein-Uhlenbeck model map, when the mean-reverting parameter θ is set to zero. In this regime, all change in the price S_t will be due to the Brownian motion dW_t, which allows for steps of any size. In this section, this motion is coarse-grained to a random walk with steps of fixed size. This allows the inputs to be fully enumerated, since the number of possible random walks with a given number of steps becomes countable and finite.

The probability versus complexity relationship is shown in Supplementary Figure 12a for all 2^{22} random walks of

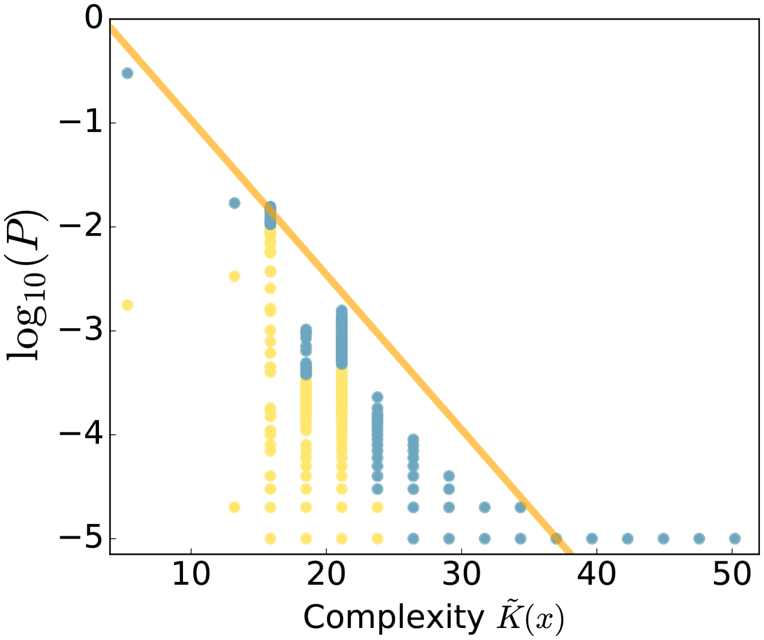

Supplementary Figure 10. The ODE cell cycle model of ref. 45, treated as an input-output map, shows simplicity bias. 10^6 inputs were randomly sampled and the output curves for the Cdc6 curve were discretised with the 'up-down' method over 40 bins. For every value of $\tilde{K}$, the blue dots represent the outputs corresponding to 50% of the inputs that produce outputs with $\tilde{K}(x) = \tilde{K}$. Since the number of outputs is too small to provide a good estimate of N_O, the upper bound line was fit to the distribution.

$m = 22$ steps. The random walk return map shows the now familiar bias towards simple outputs. Since we computed the full list of outputs by enumerating all inputs of $m = 22$ steps, we were able to calculate $\max(\tilde{K})$ and N_O directly, and find a via equation (38). It is interesting to compare the probability versus entropy plot as well, which can be seen in Supplementary Figure 12b. Here the entropy measure shows a decay which is much less pronounced than the $C_{LZ}(x)$ decay.

Given the simplicity of a random walk, this decay of the probability with complexity can also be motivated directly without needing AIT arguments. Since the random walk has no memory, every time the walker returns to $s = 0$ it can be seen as starting a new walk. In other words, the probability of any return to $s = 0$ can be represented as the product of multiple 'first return' walks. It is known[46] that the probability of first return T of a simple random walk at step m scales for large m as:

$$P_{ret}(T = m) = \frac{1}{m-1}\binom{m}{\frac{m}{2}}2^{-m} \approx \frac{1}{m-1}\frac{1}{\sqrt{\pi m/2}} \sim m^{-1.5} \tag{41}$$

where m must be an even number. Most importantly, this means that for a given number of steps, high probability outputs will be strings which have long sequences of zeros or ones, since $P(T = m)$ goes to zero relatively slowly as m increases. Similarly, the probability of a simple random walker not returning after m steps is given by[46]:

$$P(S_1 \neq 0, S_2 \neq 0, \ldots, S_m \neq 0) = 2\sum_{b=1}^{m}\frac{b}{m}P(S_m = b) \tag{42}$$

where the 2 takes into account walks in $s > 0$ and $s < 0$, and $P(S_m = b) = \binom{m}{\frac{m+b}{2}}2^{-m}$. Equation (41) implies that outputs made of simple repeated motifs will have very low probability. For instance, a run of length 6 that returns three times to the origin has a probability of $P_{ret}(T = 2)P_{ret}(T = 2)P_{ret}(T = 2) \approx (2^{-1})^3 \approx 12.5\%$, while equation (42) implies that a run of same length that does not return to 0 has a probability of 64.4%. More generally, the largest probability strings are typically ones with no or few returns, while strings with multiple returns will have low probability.

We give some examples of outputs and probabilities from the numerical simulations, enumerating all 2^{22} bitstrings. An example of the highest probability output for each unique complexity value, with format (string complexity, $\log_{10}$ probability, output string) is

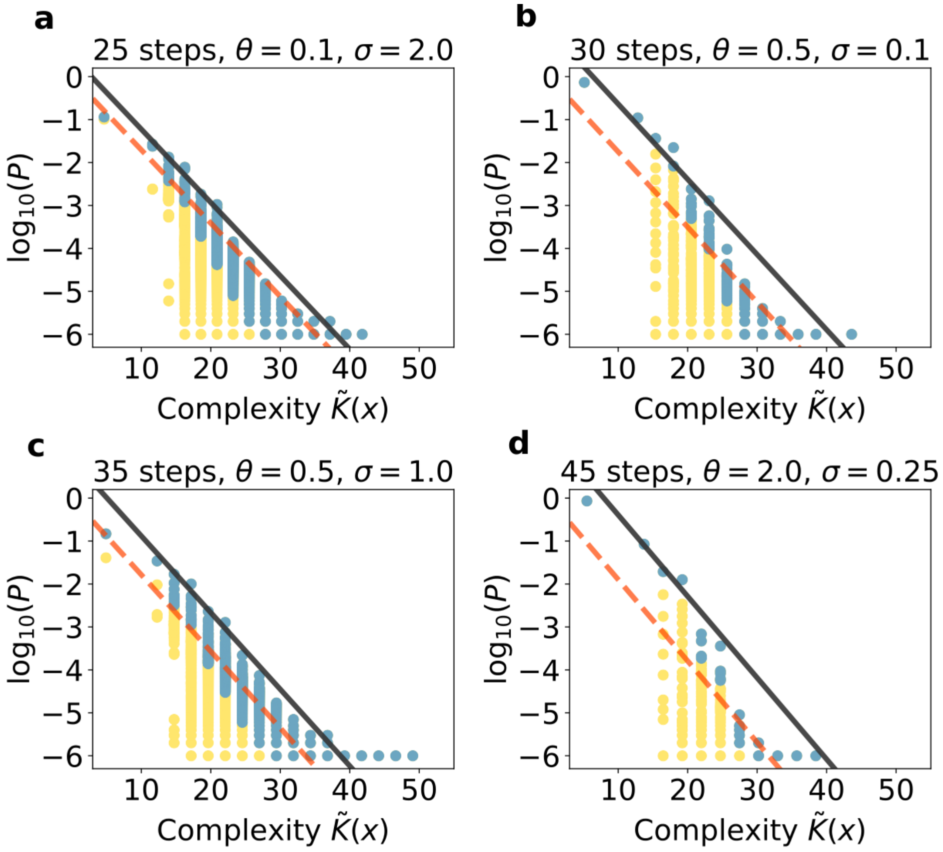

Supplementary Figure 11. The Ornstein-Uhlenbeck process shows simplicity bias for different choices of the parameters θ and σ, as well as of the number of steps of the Brownian motion. Respectively: **(a)** $\mu = 0.1$, $\sigma = 2.0$, 25 steps, a=0.56, b=-1.6, **(b)** $\mu = 0.5$, $\sigma = 0.1$, 30 steps, a=0.58, b=-3.7, **(c)** $\mu = 0.5$, $\sigma = 0.10$, 35 steps, a=0.59, b=-3.0, **(d)** $\mu = 2.0$, $\sigma = 0.25$, 45 steps, a=0.63, b=-5.0. For every value of $\tilde{K}$, the blue dots represent the outputs corresponding to 50% of the inputs that produce outputs with $\tilde{K}(x) = \tilde{K}$. Since $N_O = 2^n$, $\max(\tilde{K}(x))$ is also known, and the upper bound coefficients were calculated using equation (38). Red dashed lines represent an upper bound offset of $b = 0$.

```
4.0,  -1.08, '1111111111111111111111'
11.0, -2.40, '1111111111111111111110'
13.0, -1.66, '1100000000000000000000'
16.0, -1.66, '1011111111111111111111'
18.0, -2.24, '0011000000000000000000'
20.0, -2.24, '0100111111111111111111'
22.0, -2.81, '0100110000000000000000'
25.0, -3.39, '0011010011111111111111'
27.0, -3.82, '1011111110110000000000'
29.0, -4.22, '0100111110110000000000'
31.0, -4.78, '0100000011010011111111'
33.0, -5.32, '0100000011101100111111'
36.0, -5.84, '0100110010111110110000'
38.0, -5.92, '0100000001001110101101'
40.0, -6.62, '1011101011110000110010'
```

As expected from our back-of-the-envelope calculation for return probabilities, higher probability outputs contain long runs of zeros or ones. Further calculations in this vein could undoubtably provide more detail on the distribution of outputs. The point of this random walk illustration, however, is to demonstrate that our very simple AIT based predictions capture some of the main phenomenology without the need for detailed calculations.

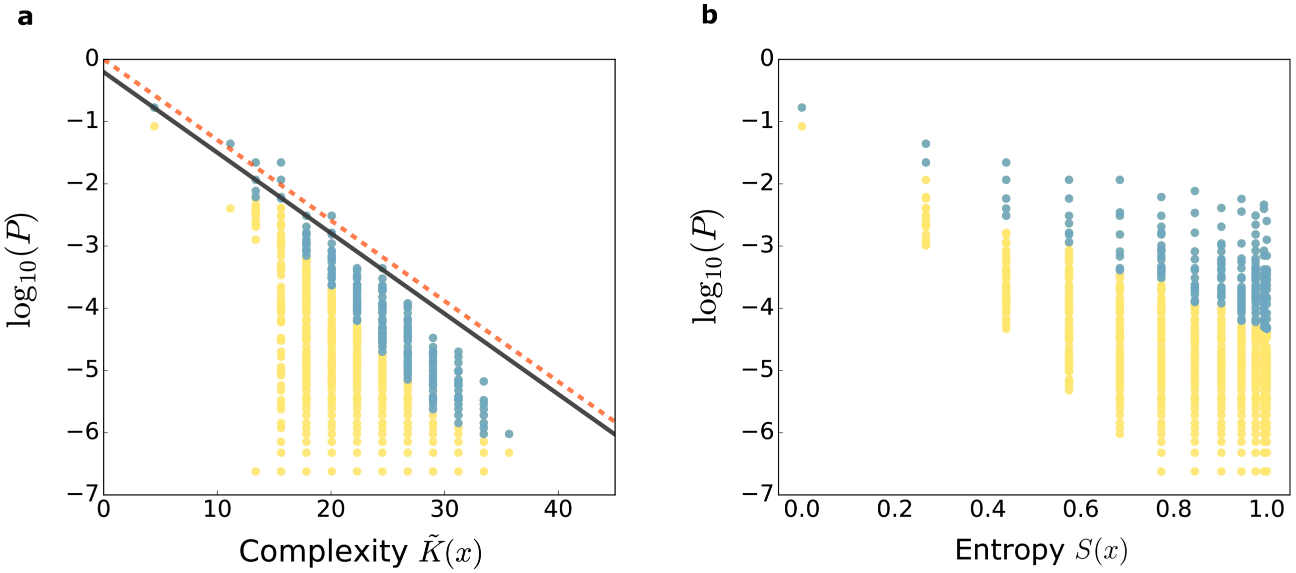

Supplementary Figure 12. Decay in probability $P(x)$ with increasing **(a)** complexity and **(b)** entropy for the random walk return map. In **(a)**, the values of $a = 0.43$ and $b = 0.68$ were estimated using the methods in Supplementary Note 8. In **(b)**, while there is a decrease in probability with increasing entropy, the upper bound for $P(x)$ does not vary in orders of magnitude as it does for $\tilde{K}(x) = C_{LZ}(x)$. For every value of $\tilde{K}$, the blue dots represent the outputs corresponding to 50% of the inputs that produce outputs with $\tilde{K}(x) = \tilde{K}$.

Polynomial curves

In this section, we study a particularly simple map. Inputs are the coefficients α_i of a polynomial $y(t) = \sum_{i=0}^{n} \alpha_i t^i$ and outputs are the resulting curve over the variable t. We show that this input-output map also exhibits bias towards simple outputs. As with the ODE systems above, this input-output map goes from the continuous space of all n-tuples of real coefficients, i.e. $\mathbb{R}^n$, to the also continuous space of all polynomial curves $y(t)$. Consequently the inputs cannot be fully enumerated, and every output curve is unique. It is therefore necessary to coarse-grain both the input and output spaces.

The space of possible polynomials is extremely large. To simplify, we take the coefficients α_i from a normal distribution of mean zero and variance one. Polynomials of this form are also known as Kac polynomials[47]. It is known that the expected number of real roots for a Kac polynomial of degree n is proportional to $\log n$[48], but the actual distribution of the number of zeros does not have a known closed formula. One advantage of Kac polynomials is that they have symmetry properties that lead to some simplifications: Since the distribution of coefficients α_i is symmetric around zero, for every positive root there should be a negative root, which means that the negative real roots should be distributed in the same way as their positive counterparts. Second, for every polynomial $p(x)$ with coefficients $(a_0, a_1, \ldots, a_{n-1}, a_n)$ there will be a polynomial $\tilde{p}(x)$ with the same coefficients, but in the reverse order: $(a_n, a_{n-1}, \ldots, a_1, a_0)$. For every $x \neq 0$ which is a root of $p(x)$, the reverse polynomial $\tilde{p}(x)$ will have a root at $1/x$. Due to this property, we only need to evaluate $y(t)$ in $[0, 1]$, since for every root in this interval there will be a root in $[1, \infty)$, in addition to their negative mirror images. In summary, because of these symmetries, the outputs can be considered in $[0, 1]$ only.

We coarse-grain the outputs in two ways. In the first one, we used the 'up-down' method described above, thus converting every output into a binary output which we here call *string 1*. And since the 'up-down' method produces a string that captures the changes in sign of the derivative of $y(t)$, i.e. the roots of its derivative, we also produced a binary string simply describing the location of the roots of $y(t)$. *string 2* is made entirely of 0s, except for the digits representing the intervals where $y(t)$ crosses zero, which are 1s. For example, if $y(t)$ crosses zero three times in three different discrete bins, then its corresponding output string will then have three 1s, while if $y(t)$ does not cross zero it will correspond to a string made only of 0s.

One problem that remains is that since we don't know the exact distribution of roots in advance, there could be multiple roots in a single bin when roots are closer than the distance between bins. This is hard to completely rule out. Instead, since the goal in this section is to simply demonstrate the existence of simplicity bias in this input-output map, we simply check for the severity of this problem by varying the number of bins in the discretisation, showing

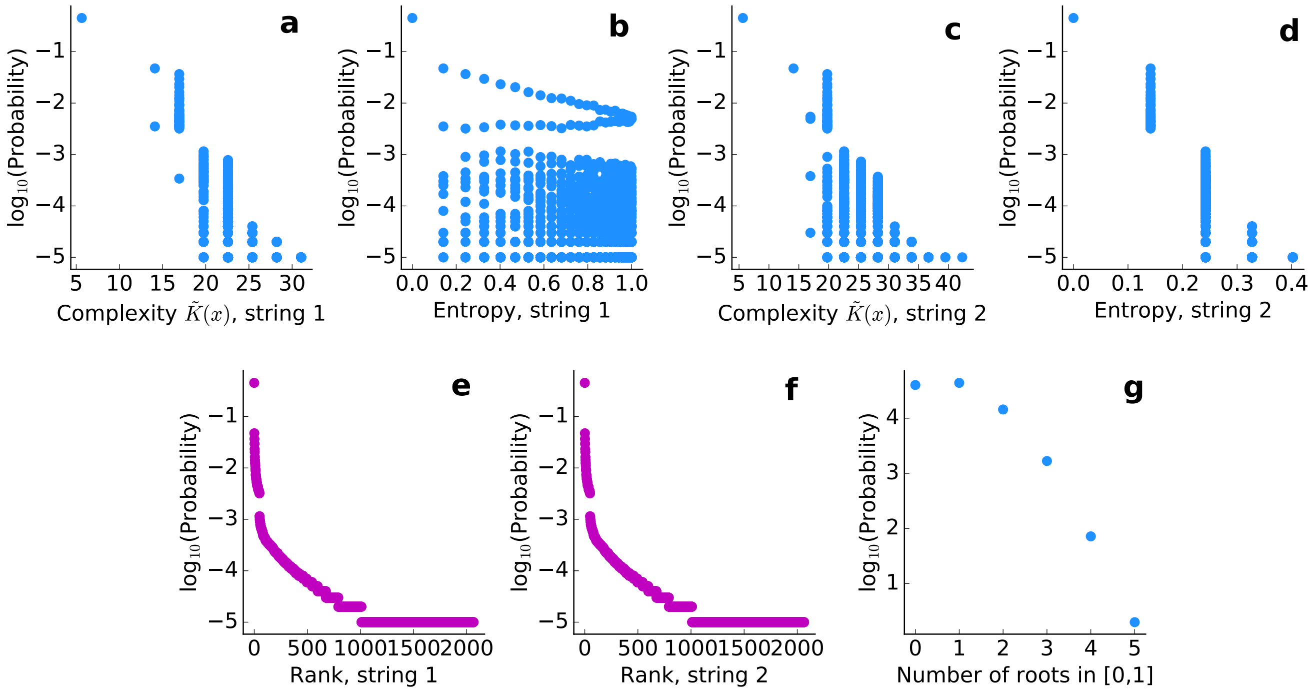

Supplementary Figure 13. Simplicity bias in a simple polynomial input-output map. **(a)** and **(b)** respectively show output probability versus output complexity, and output probability versus output entropy, for outputs defined as *string 1*, which indicates changes of sign of the slope. Similarly, **(c)** and **(d)** show probability versus complexity and probability versus entropy, for outputs defined as *string 2*, which indicates the coarse-grained location of the roots of a given polynomial. **(e)** and **(f)** show the probability of all output strings 1 and 2 respectively, from most probable to least probable output. Note that despite *string 1* and *string 2* being defined differently, both present the same decay in **(e)** and **(f)**. Finally, **(g)** shows the distribution of the number of roots in the $[0, 1]$ interval. Polynomials of degree 10 were used for all figures, and the $[0, 1]$ interval was divided in 50 bins $(t, t + \delta t)$, where $\delta t = 0.02$.

that the simplicity bias phenomenology is present for different bin sizes.

To proceed we take input coefficients from a standard normal distribution. The resulting polynomials are evaluated only in $[0, 1]$ and then discretised into binary strings using the methods above. We sampled 10^5 polynomials of degree $d = 10$, discretising their outputs into 50 bit-long strings, and comparing the complexity $\tilde{K}(x)$ and the entropy $S(x)$ of an output to the number of inputs corresponding to each output, i.e. the probability of that output.Despite being very simple and coarse-grained, this input-output map shows the basic simplicity bias phenomenology we predicted above. As seen in Supplementary Figures 13a and 13c, random sampling of inputs (coefficients) is much more likely to produce low complexity curves than more complex curves.

Supplementary Figure 13 also shows other proxies for output complexity. For example, Supplementary Figure 13b compares the probability of output *string 1* with its entropy $S(x)$. In sharp contrast to the complexity $\tilde{K}(x)$, there is no clear relationship between the probability and the $S(x)$. The reason for this is not hard to work out. Entropy is maximal for an equal number of one's and zero's, and minimal when either one or zero dominates. Thus simple patterns like 01010101010101010101 or 11111111110000000000 have low complexity when measured by $C_{LZ}(x)$ but maximal entropy. For *string 1* many outputs of varying entropy will correspond to similar polynomial curves. For example, if the derivative of a polynomial has a single root in $[0, 1]$, the proportion of 0s and 1s in *string 1* will depend on where the root is located, since *string 1* indicates where $y(t)$ is increasing or decreasing. This leads to nearly identical outputs – curves with a single extremum – having very different values for entropy. On the other hand, as can be seen in Supplementary Figures 13c and 13d for *string 2* both measures of complexity show a clear correlation. In this case, the complexity $\tilde{K}(x)$ and the entropy $S(x)$ of the output string, are essentially proxies for the number of roots in $[0, 1]$, which drops quickly with increasing number of roots, as shown in Supplementary Figure 13g.

Both output definitions, *string 1* and *string 2*, give rise to the same distribution of probability per output, shown by the rank plot in Supplementary Figures 13e and 13f. Supplementary Figure 13g shows the distribution of the number of roots in $[0, 1)$,

This bias towards simple outputs is observed for polynomials of varying degree, with varying levels of discretisation.

In Supplementary Figures 14 and 15 we show that our results are robust to different levels of discretisation of the outputs, as well as to different polynomial degrees. For such a simple system it is possible to rationalise this bias in other ways as well, but the point of this exercise is to show that a simple application of our simplicity bias arguments seems to capture some of the dominant aspects of the bias.

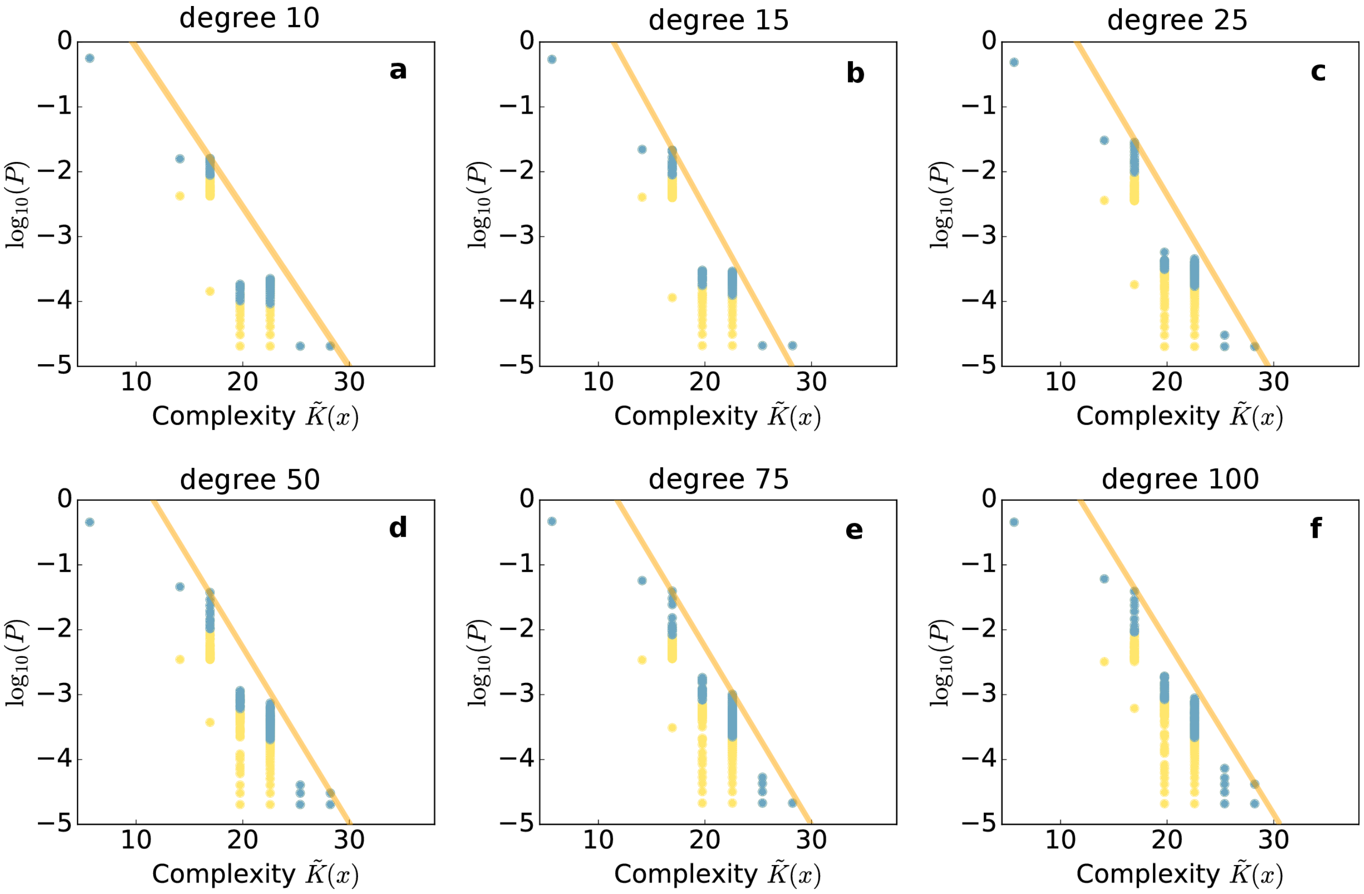

Supplementary Figure 14. Graphs showing probability versus complexity of an output string, for polynomials of varying degrees and fixed discretisation (50 bins). Panels **(a)** to **(f)** represent polynomials of degree 10, 15, 25, 50, 75 and 100 respectively. Each plot was produced from a sample of 10^5 polynomials. For every value of $\tilde{K}$, the blue dots represent the outputs corresponding to 50% of the inputs that produce outputs with $\tilde{K}(x) = \tilde{K}$. Since the number of outputs is too small to provide a good estimate of N_O, the upper bound line was fit to the distributions.

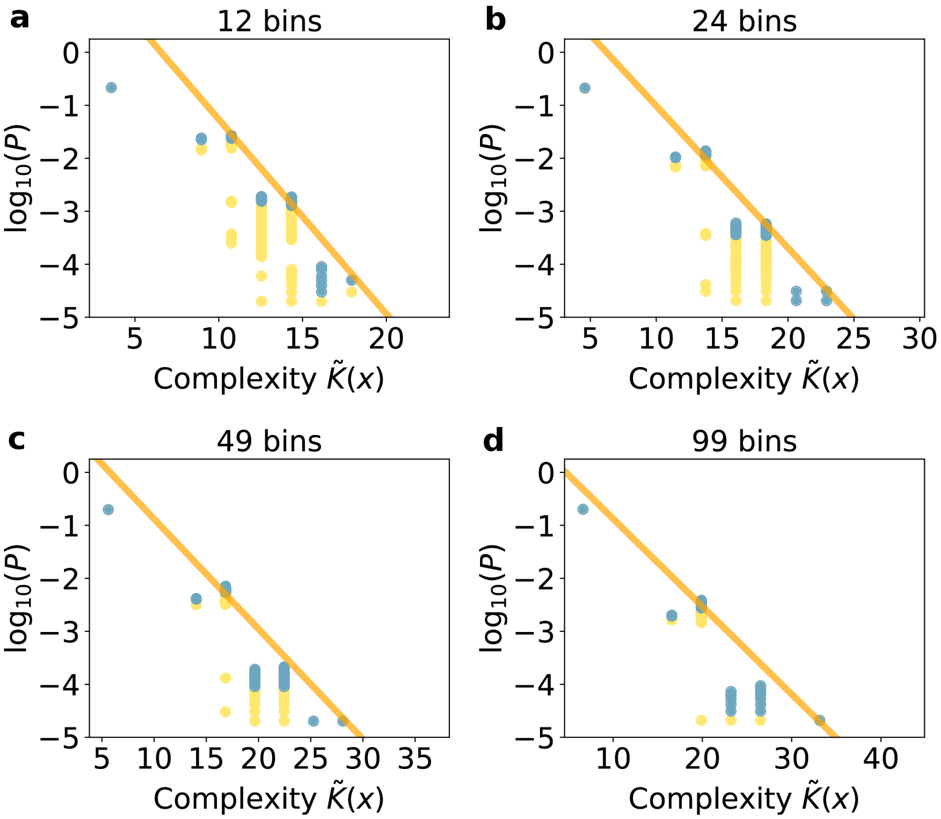

Supplementary Figure 15. Graphs showing probability versus complexity of an output string, for polynomials of varying discretisation and fixed degree ($d = 14$). Panels **(a)** to **(d)** represent polynomials discretised in 12, 24, 49 and 99 bins respectively. Each plot was produced from a sample of 10^5 polynomials, and for every value of $\tilde{K}$, the blue dots represent the outputs corresponding to 50% of the inputs that produce outputs with $\tilde{K}(x) = \tilde{K}$. Since the number of outputs is too small to provide a good estimate of N_O, the upper bound line was fit to the distributions.

The matrix map: how map complexity affects simplicity bias

In the main text we discussed a matrix map illustrated in Supplementary Figure 16. Here the inputs are binary vectors p of length n and the outputs are binary vectors x of length n. The outputs are produced by first performing a simple matrix multiplication $z = M \cdot p$, where M is an $n \times n$ matrix, and then applying a thresholding function such that each element z_i is transformed to the outputs vector x such that the output is 0 if $z_i < 0$ and 1 if $z_i \geq 0$.

The reason for the threshold function is to ensure the nonlinearity of this input-output map. A linear map, such as $z = M \cdot p$, is incapable of producing bias towards any output, since it is simply an injective function from $\mathbb{R}^n$ into a subset $X \subseteq \mathbb{R}^n$, and two different inputs cannot produce the same output. The threshold function thus forces the map to have some degeneracy.

Here we treat this map as a very general input-output system. However, this matrix map can also be seen as simple a neural network, made only by the input and output layers, each with n neurons, having a step function as its activation function, and no hidden layers. Although this perspective is not explored in this paper, it is interesting to note that thinking of a neural network as a matrix map translates the machine learning task of finding a set of weights that minimises a cost function to the task of finding an input-output map with certain desired properties. A similar system was used as a very simple model of transcriptional gene networks in ref. 49, where it was shown that this map can generate biased outputs.

The space of possible matrices is very large, so we begin with a very simple random 20×20 matrix where every entry is chosen from $\{-1, 1\}$ with uniform probability. Supplementary Figure 17a shows that this map generates a very biased distribution of inputs over outputs, but, in sharp contrast to the other systems studied in this paper, there is no bias towards simpler or more complex outputs, as it can be seen in Supplementary Figure 17b.

To show that most maps have a bias in their input-output maps, we use a very simple bias ratio measure, first introduced in ref. 41. If one takes the entropy of the distribution of the probabilities p_i of the N_O outputs, $H =$

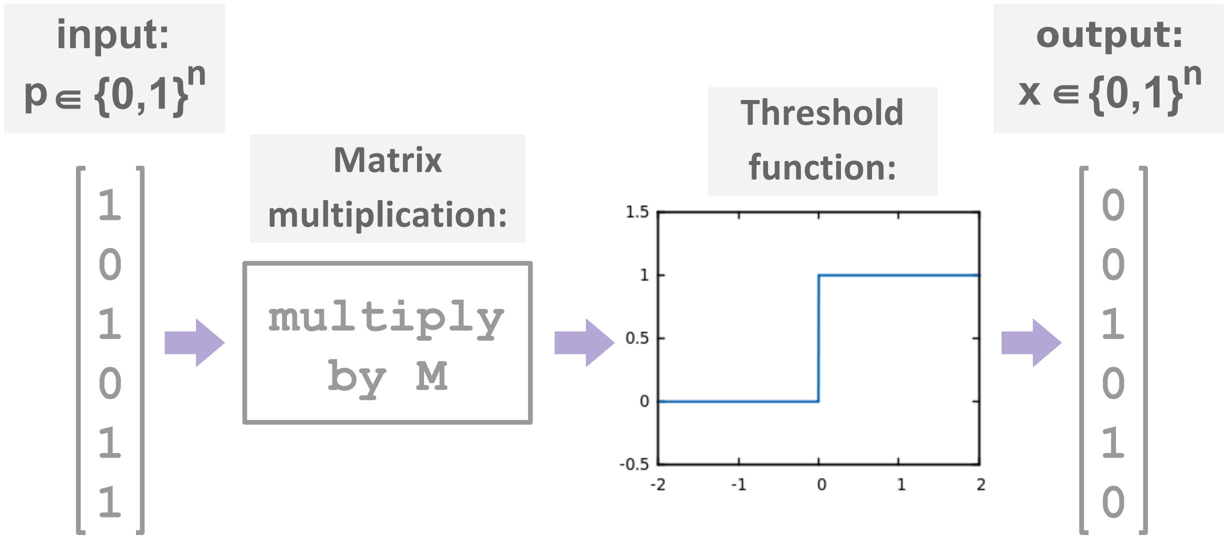

Supplementary Figure 16. Illustration of an input-output map consisting in binary vectors multiplied by a matrix, following by a binary threshold so that the output also consists in binary vectors.

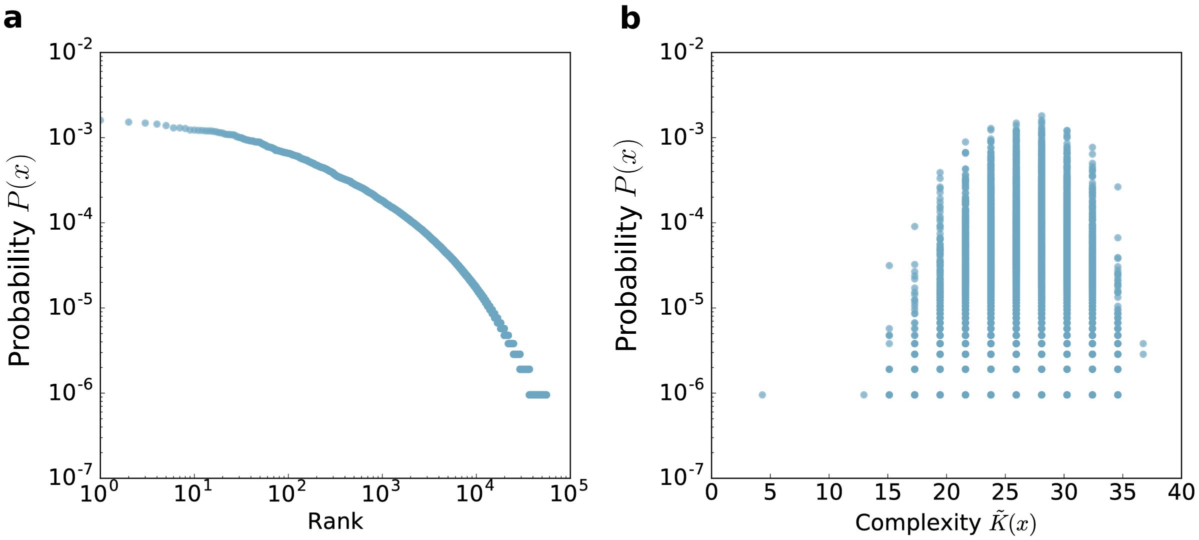

Supplementary Figure 17. Properties of a matrix map made from a random 20×20 matrix where every entry is chosen from $\{-1, 1\}$ with uniform probability. **(a)** A rank plot shows that the map exibhits bias in that certain outputs are much more likely to occur than others. **(b)** However, in contrast to simplicity bias phenomenology, there is no clear correlation between probability and complexity of the output.

$-\sum_{i=1}^{N_O} p_i \log_2 p_i$, then the exponential of the entropy, 2^H, should be a rough estimate of the effective number of outputs[50]. One can then define a *bias ratio* $\beta = 2^H/N_O$, which measures the ratio between the effective number of outputs and the total number of outputs in that map. For maps with $\beta \approx 1$, inputs should be roughly uniformly distributed across outputs, while for maps with $\beta \ll 1$, most inputs should correspond to a few outputs. In other words, the closer β is to 0, the stronger the bias in the distribution of inputs over outputs. For example, the map in Supplementary Figure 17 has a bias parameter $\beta = 0.56$. In Supplementary Figure 18a we show a histogram produced from an ensemble of 5000 matrices where each of the 20×20 entries was chosen uniformly from $\{-1, 1\}$. We observe that most matrix maps in that ensemble have small β, and so most inputs map to a small fraction of the outputs. The maps show bias.

For this same set of 5000 matrices we calculated the probability that a given output string would appear. We then measured the complexity of each output, and generated a distribution of outputs probability versus complexity. As can be seen in Supplementary Figure 18b, the distribution of these strings is very close to what we would expect

by random sampling of the outputs, or in fact random sampling of all binary strings of length 20. We also sampled over 5000 random 20×20 matrices where every entry is taken from a standard normal distribution ($\mu = 0, \sigma = 1$), finding very similar results. We distinguish in the plot between input sampling and output sampling. The former takes averages over outputs generated by uniform random sampling of inputs that are fed into the map, and the latter simply samples uniformly over the outputs found. For an individual matrix with bias, input and output sampling can vary significantly, depending on the amount of bias. However, when we subsequently average over the matrices, this difference goes away. For the matrices with randomly chosen $\{-1, 1\}$ entries, there remains a very slight difference between input and output sampling, while for the matrices with entries taken from a standard normal distribution input and output sampling yield essentially the same results within our measurement errors. In other words, while for a single matrix with bias, certain outputs are much more likely to be generated by random sampling of inputs than others, when averaged over many matrices, no output string is more or less likely to be generated than any other. This behaviour is fundamentally different from maps that show simplicity bias, because even if we averaged over maps, we would expect low complexity outputs strings to be more likely to occur on average.

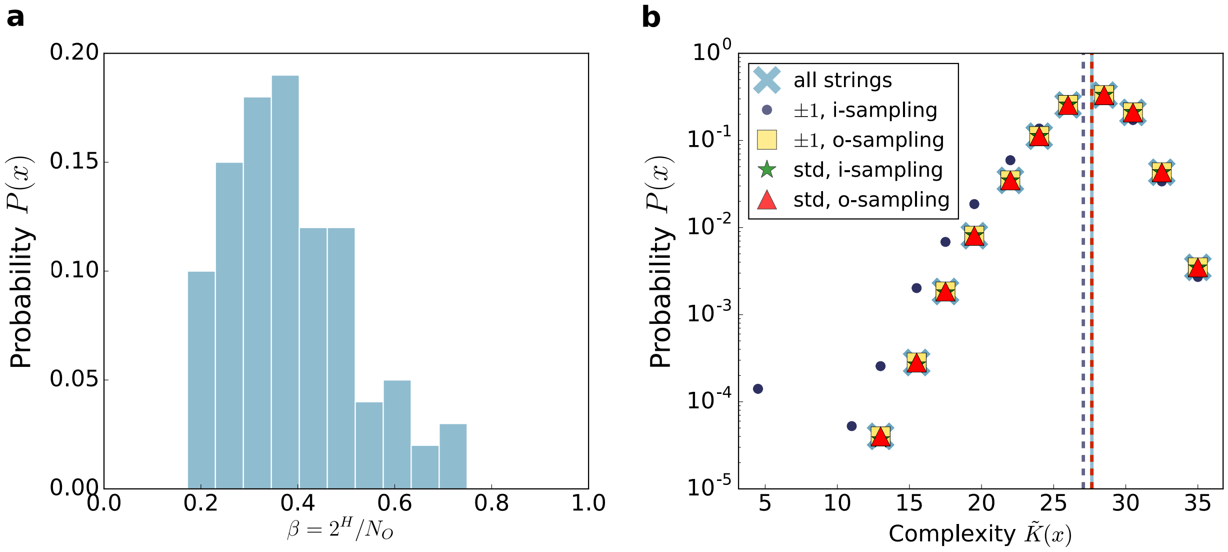

Supplementary Figure 18. **(a)** Histogram for the bias ratio β, a measure for the ratio between the number of effective outputs and the total number of outputs of a map. Smaller values of β mean more bias in the map. The map in Supplementary Figure 17 has a bias parameter $\beta = 0.56$. **(b)** Distribution of $\tilde{K}(x)$ for the outputs of matrix maps from an ensemble of random 20×20 matrices with entries chosen uniformly from $\{-1, 1\}$. Dark blue circles denote a distribution made by sampling inputs and yellow squares denote a distribution made by uniformly sampling over outputs, The green stars and red triangles respectively represent the same input-sampled and output-sampled averages, but for random 20×20 matrices with taken from a standard normal distribution ($\mu = 0, \sigma = 1$). The light blue crosses represent the distribution of $\tilde{K}(x)$ over all bitstrings of length 20. The overlap between all curves shows that the outputs of a random matrix map are likely to be as complex as a random set of strings of the same length. Error bars are too small to be visible, and average values are all within 27.3 ± 0.3.

Circulant matrices

All the matrix maps considered so far were made from random matrices, with entries either taken from $\{-1, 1\}$ or from a normal distribution of mean zero and variance 1. In other words, for square matrices, the amount of information needed to specify the map grows as n^2. In this section we study a set of matrix maps for which the amount of information needed to specify the map grows more slowly with n. We begin by studying circulant matrices, illustrated in Supplementary Figure 19a. Circulant matrices are a class of Toeplitz matrices where each row corresponds to the row above, shifted to the right by one element. They play a role in fields ranging from discrete Fourier transforms to cryptography[51]. In this work, their most important aspect is that these matrices are defined by the values on the first row. Limiting our matrix entries to $\{-1, 1\}$, a circulant matrix can be defined using n bits or less, as opposed to n^2 bits for a random $\{-1, 1\}$ matrix. These matrices are illustrated in Supplementary Figure 19a.

While the outputs from the random matrix maps explored in the previous section were, on average, as complex as any random binary string of the same length, some circulant matrix maps do show some bias towards simple outputs (Some fully random maps may also show simplicity bias, but these are sufficiently rare that we do not find any in our

sampling). Supplementary Figure 19c, shows the ratio between $\tilde{K}_o$, the average output complexity when all outputs are assigned the same weight, and $\tilde{K}_i$, the average output complexity where every output is weighed by the frequency with which it appears upon random sampling of inputs. The majority of maps have $\tilde{K}_o/\tilde{K}_i$ close to one, but a small fraction (a few %) have significantly higher ratios, which mean that low complexity outputs are more likely to appear upon random sampling of inputs.

The main correlating factor for matrices that have high values of $\tilde{K}_o/\tilde{K}_i$ is that the complexity $\tilde{K}(row)$ of the top row which determines the whole matrix is low. We showed this in the main text in Figure 2, which is repeated above as Supplementary Figure 19d for clarity. This behaviour is what we would expect from our general derivation of simplicity bias, which relies on the map itself being having a limited complexity.

To check that this bias towards simpler output presented by a small fraction of the circulant matrix maps could not be attributed to the matrices having low rank we also measure the rank of each matrix. As shown in Supplementary Figure 19b for an ensemble of 25000 circulant matrices with 20×20 entries taken from $\{-1, 1\}$, most matrices have a matrix rank close to its maximum of 20, with 93.8% of the matrices having a rank of 20. We also confirmed that the matrices with high values of $\tilde{K}_o/\tilde{K}_i$ do not have low rank on average.

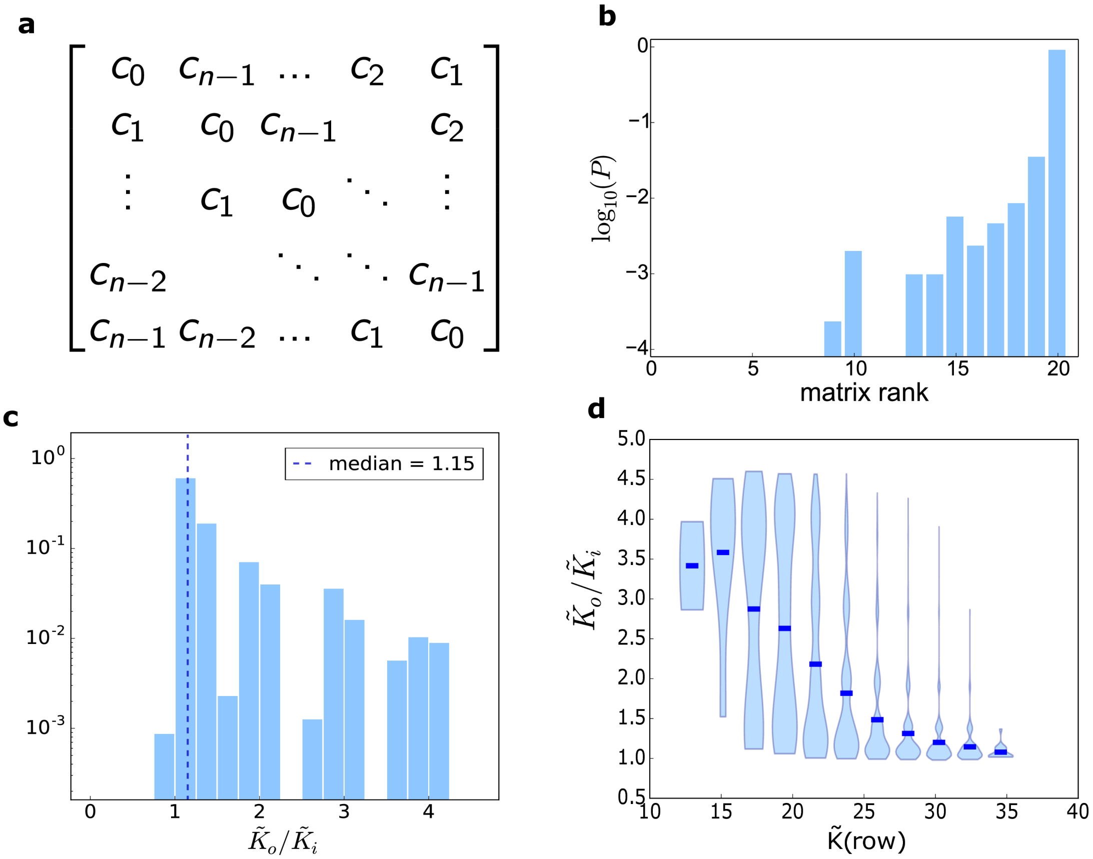

Supplementary Figure 19. **(a)** Illustration of a circulant matrix. **(b)** Histogram for the matrix rank of 25000 circulant matrices with 20×20 entries taken from $\{-1, 1\}$. **(c)** Histogram for the ratio $\tilde{K}_o/\tilde{K}_i$ in the matrix maps made from the same matrices. **(d)** Violin plots showing how $\tilde{K}_o/\tilde{K}_i$ changes with the complexity of the top row of the same circulant matrices. Also shown as Figure 2 in the main text.

Low complexity circulant matrices show simplicity bias

We can explore this behaviour in more detail by creating circulant matrices with low complexity rows. We limited the $\{-1, 1\}$ matrix ensemble to 20×20 circulant matrices defined by rows containing $\log n$ positive entries (and negative entries otherwise). Those matrices, by construction, are defined by $\mathcal{O}(\log^2 n)$ bits, a much smaller amount of information when compared to the $n \times n$ bits required to specify each entry of a random matrix with entries taken randomly from $\{-1, 1\}$, or the n bits to define most circulant matrices in the full ensemble (Note that most strings

of length n have (near) maximum (or modal for C_{LZ}) complexity). For the matrix maps made from these simpler matrices, the evidence for simplicity bias is clear: all maps show a ratio $\tilde{K}_o/\tilde{K}_i > 3.5$.

Since those matrices will consist in 320 entries equal to -1 and 80 entries equal to $+1$, we ran a control using using matrices with these same proportions, but without the row-by-row structure, as well as matrices with the same proportion of $+1$ and -1 per row $(16:4)$, but without the Toeplitz structure. We tested both alternatives, but none showed simplicity bias as the simple matrices described in this section do. This can be attributed to the fact that these maps need not $\mathcal{O}(\log^2 n)$ bits to be described, but $\mathcal{O}(n \log n)$, since all row structure is lost. They thus violate our limited complexity condition that $K(f) + K(n) \ll K(x)$.

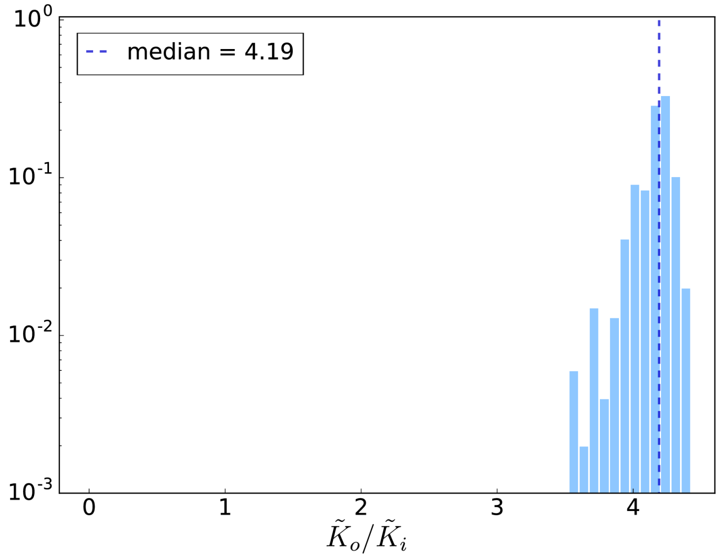

Supplementary Figure 20. Histogram for $\tilde{K}_o/\tilde{K}_i$ for 20×20 circulant matrices with entries taken from $\{-1, 1\}$, containing only $\lfloor \log 20 \rfloor = 4$ positive entries per row.

Rank and sparsity do not explain simplicity bias in the matrix map

Rank and sparsity are two traditional measures for the complexity of matrices. As another control, we consider 10×10 matrices of rank and sparsity varying from 1 to their maximum values of 10 and 100 respectively.

Firstly, in Supplementary Figure 21 we show violin plots for the output complexity of random 10×10 matrix maps made from random $\{-1, 1\}$ matrices versus rank. As the matrix rank decreases from 10 to 1, both $\tilde{K}_o$ and $\tilde{K}_i$ deviate from the full-rank matrices. Supplementary Figure 21c, however, shows that the ratio $\tilde{K}_o/\tilde{K}_i$ stays constant. In other words, even though matrix maps made from matrices of lower rank do produce low complexity outputs, that is not because those outputs correspond to more inputs than their high complexity counterparts, but rather because the high complexity outputs are not being produced at all. In an extreme case, a matrix of rank one will produce the simplest possible outputs, but nothing more than that.

The second natural candidate for the complexity of a matrix map is the sparsity of its matrix, i.e. the number of zeros in the matrix. As shown in Supplementary Figure 22 for 10×10 matrices, $\tilde{K}_o$ and $\tilde{K}_i$ remain the same for levels of sparsity up to 80%, and drop sharply for sparser matrices. Still, the ratio $\tilde{K}_o/\tilde{K}_i$, remains around 1, indicating once again a decrease in output complexity but no bias towards producing simpler outputs over more complex outputs.

In summary, even though rank and sparsity are used as proxies for matrix complexity in other contexts, we have presented evidence that they only affect the average output complexity of a matrix map when at extreme values of either low rank or high sparsity. Even in these cases, the ratio $\tilde{K}_o/\tilde{K}_i$ remains around 1. This means that within all the outputs produced by these low-rank or high-sparsity matrix maps, there is no bias towards producing simpler outputs with a higher probability .

On the other hand, we have shown that one can define simple matrix maps without resorting to low-rank or sparse matrices, by using circulant $\{-1, 1\}$ matrices with a small number of positive entries on each row - approximately $\log n$ elements on a $n \times n$ matrix. And as mentioned above, the matrix maps from this ensemble can be defined with $\mathcal{O}(\log^2 n)$ bits of information. While in the main text and in Supplementary Note 3 we mainly treat maps of

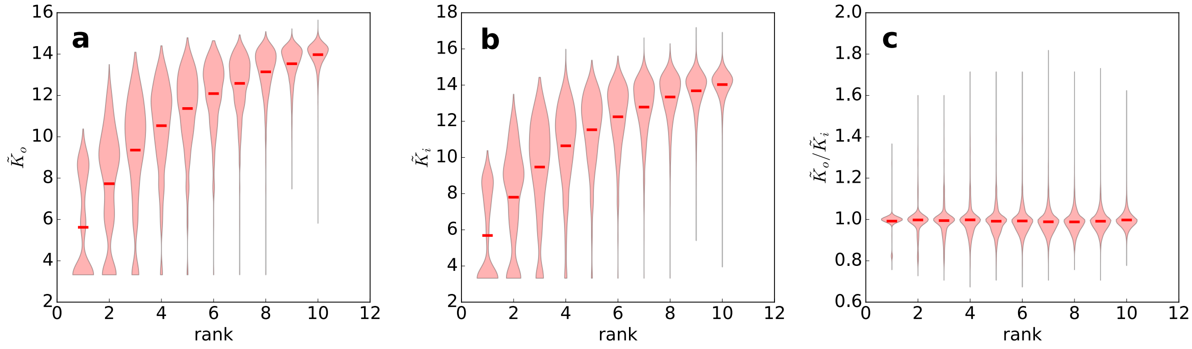

Supplementary Figure 21. Violin plots for the complexity of random $\{-1, 1\}$ matrix maps made from 10×10 matrices as a function of rank. $\tilde{K}_o$ in **(a)** shows the average output complexity, while $\tilde{K}_i$ in **(b)** shows the average output complexity where each output is weighed by its frequency, i.e. the fraction of inputs that correspond to it. Figure **(c)** shows the ratio $\tilde{K}_o/\tilde{K}_i$. Red lines mark the average values in all violin plots.

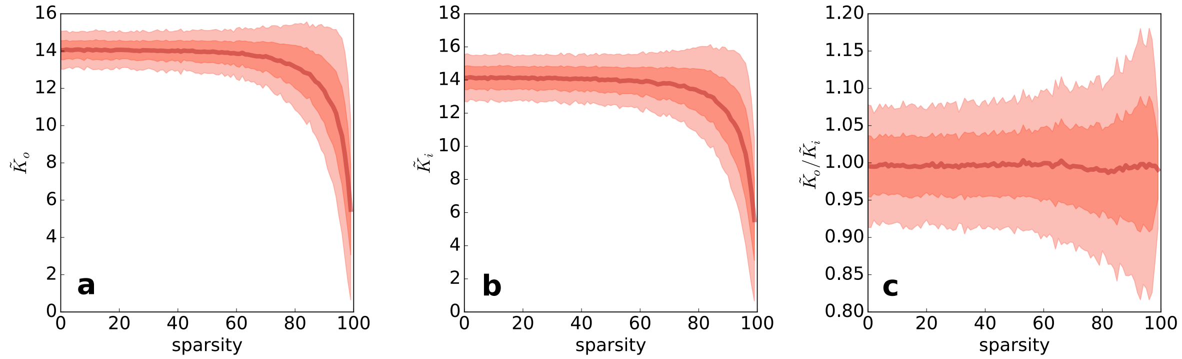

Supplementary Figure 22. Output complexity of random $\{-1, 1\}$ matrix maps made from 10×10 matrices, versus matrix sparsity. $\tilde{K}_o$ in **(a)** shows the average output complexity, while $\tilde{K}_i$ in **(b)** shows the average output complexity where each output is weighed by its frequency, i.e. the fraction of inputs that correspond to it. Panel **(c)** shows the ratio $\tilde{K}_o/\tilde{K}_i$. The darkest red lines represent the average $\tilde{K}_o$, $\tilde{K}_i$ and their ratio $\tilde{K}_o/\tilde{K}_i$, and the lighter shades of red represent one and two standard deviations.

fixed complexity, if maps grow only as $\log^2 n$ then for large enough n the requirement that $K(f) \ll K(x)$ should always hold. However, we only tried an extremely small range of $\log n$ so these conclusions are very preliminary. Again, as discussed earlier, we find clear simplicity bias phenomenology even when we may not quite be in the limit of $K(f) \ll K(x)$. This suggests that the large n asymptotic behaviour persists down to smaller maps. We have not yet explored how small the matrix maps should be for finite size effects, discussed for example in Supplementary Note 9, to start kicking in.

---

[1] M. Li and P.M.B. Vitanyi. *An introduction to Kolmogorov complexity and its applications.* Springer-Verlag New York Inc, 2008.

[2] C.S. Calude. *Information and randomness: An algorithmic perspective.* Springer, 2002.

[3] P. Gács. *Lecture notes on descriptional complexity and randomness.* Boston University, Graduate School of Arts and Sciences, Computer Science Department, 1988.

[4] K.F. Riley, M.P. Hobson, and S.J. Bence. *Mathematical methods for physics and engineering.* Cambridge University Press, 2006.

[5] Alan Mathison Turing. On computable numbers, with an application to the entscheidungsproblem. *J. of Math*, 58(345-363):5, 1936.

[6] I.L. Hofacker, W. Fontana, P.F. Stadler, L.S. Bonhoeffer, M. Tacker, and P. Schuster. Fast folding and comparison of RNA secondary structures. *Monatshefte für Chemie/Chemical Monthly*, 125(2):167–188, 1994.

[7] Seth Lloyd. Quantum-mechanical computers and uncomputability. *Physical review letters*, 71(6):943, 1993.
[8] Toby S Cubitt, David Perez-Garcia, and Michael M Wolf. Undecidability of the spectral gap. *Nature*, 528(7581):207–211, 2015.
[9] R. J. Solomonoff. A preliminary report on a general theory of inductive inference (revision of report v-131). *Contract AF*, 49(639):376, 1960.
[10] A.N. Kolmogorov. Three approaches to the quantitative definition of information. *Problems of information transmission*, 1(1):1–7, 1965.
[11] G.J. Chaitin. *Algorithmic information theory.* Cambridge University Press, 1987.
[12] Gregory J Chaitin. A theory of program size formally identical to information theory. *Journal of the ACM (JACM)*, 22(3):329–340, 1975.
[13] Peter Grunwald and Paul Vitányi. Shannon information and Kolmogorov complexity. *arXiv preprint cs/0410002*, 2004.
[14] L.A. Levin. Laws of information conservation (nongrowth) and aspects of the foundation of probability theory. *Problemy Peredachi Informatsii*, 10(3):30–35, 1974.
[15] TM Cover and J.A. Thomas. *Elements of information theory.* John Wiley and Sons, 2006.
[16] The $K(f)$ that is relevant in this case is not the complexity of the full Vienna package, but simply the minimal amount of information needed to find the lowest free-energy secondary structure for a given sequence. The amount of information may be still be rather small, as it only needs as input thermodynamic model used, which is quite simple, combined with some way to search over all possible structures. In contrast to the Vienna package, for example, computing speed is not important. Nevertheless, it is likely that $K(f)$ will be larger than the complexity of many simple $L = 20$ outputs which can be described in less than 32 bits).
[17] Walter Rudin. *Functional analysis.* Tata McGraw-Hill, 1991.
[18] Markov's inequality is a standard result: if z is nonnegative random variable distributed according to a probability distribution $P(z)$, then $P(z > a) < E_z/a$, where E_z is the expected value of z given $P(z)$. If $\bar{z} = z/E_z$ then $P(\bar{z} > r) < 1/r$.
[19] F. Kaspar and HG Schuster. Easily calculable measure for the complexity of spatiotemporal patterns. *Physical Review A*, 36(2):842, 1987.
[20] Thomas Schürmann and Peter Grassberger. Entropy estimation of symbol sequences. *Chaos: An Interdisciplinary Journal of Nonlinear Science*, 6(3):414–427, 1996.
[21] E. Rivals, O. Delgrange, J.P. Delahaye, M. Dauchet, M.O. Delorme, A. Hénaut, and E. Ollivier. Detection of significant patterns by compression algorithms: the case of approximate tandem repeats in DNA sequences. *Computer applications in the biosciences: CABIOS*, 13(2):131–136, 1997.
[22] P. Ferragina, R. Giancarlo, V. Greco, G. Manzini, and G. Valiente. Compression-based classification of biological sequences and structures via the universal similarity metric: experimental assessment. *BMC bioinformatics*, 8(1):252, 2007.
[23] M. Li, J.H. Badger, X. Chen, S. Kwong, P. Kearney, and H. Zhang. An information-based sequence distance and its application to whole mitochondrial genome phylogeny. *Bioinformatics*, 17(2):149–154, 2001.
[24] X. Chen, B. Francia, M. Li, B. Mckinnon, and A. Seker. Shared information and program plagiarism detection. *Information Theory, IEEE Transactions on*, 50(7):1545–1551, 2004.
[25] R. Cilibrasi, P. Vitányi, and R. Wolf. Algorithmic clustering of music based on string compression. *Computer Music Journal*, 28(4):49–67, 2004.
[26] Annick Lesne, Jean-Luc Blanc, and Laurent Pezard. Entropy estimation of very short symbolic sequences. *Physical Review E*, 79(4):046208, 2009.
[27] H. Zenil and J.P. Delahaye. An algorithmic information theoretic approach to the behaviour of financial markets. *Journal of Economic Surveys*, 25(3):431–463, 2011.
[28] Coco Krumme, Alejandro Llorente, Manuel Cebrian, Esteban Moro, et al. The predictability of consumer visitation patterns. *Scientific reports*, 3, 2013.
[29] Paul MB Vitányi. Similarity and denoising. *Philosophical Transactions of the Royal Society A: Mathematical, Physical and Engineering Sciences*, 371(1984), 2013.
[30] Annick Lesne. Shannon entropy: a rigorous notion at the crossroads between probability, information theory, dynamical systems and statistical physics. *Mathematical Structures in Computer Science*, 24(03):e240311, 2014.
[31] C. Adami and N. J. Cerf. Physical complexity of symbolic sequences. *Physica D*, 137(1):62–69, 2000.
[32] A. Lempel and J. Ziv. On the complexity of finite sequences. *Information Theory, IEEE Transactions on*, 22(1):75–81, 1976.
[33] Jacob Ziv and Abraham Lempel. Compression of individual sequences via variable-rate coding. *IEEE transactions on Information Theory*, 24(5):530–536, 1978.
[34] José M Amigó, Janusz Szczepański, Elek Wajnryb, and Maria V Sanchez-Vives. Estimating the entropy rate of spike trains via lempel-ziv complexity. *Neural Computation*, 16(4):717–736, 2004.
[35] E Estevez-Rams, R Lora Serrano, B Aragón Fernández, and I Brito Reyes. On the non-randomness of maximum lempel ziv complexity sequences of finite size. *Chaos: An Interdisciplinary Journal of Nonlinear Science*, 23(2):023118, 2013.
[36] Jacob Ziv and Abraham Lempel. A universal algorithm for sequential data compression. *IEEE Transactions on information theory*, 23(3):337–343, 1977.
[37] J.P. Delahaye and H. Zenil. Numerical evaluation of algorithmic complexity for short strings: A glance into the innermost structure of algorithmic randomness. *Appl. Math. Comput.*, 219:63–77, 2012.
[38] Hector Zenil, Fernando Soler-Toscano, Jean-Paul Delahaye, and Nicolas Gauvrit. Two-dimensional Kolmogorov complexity and an empirical validation of the coding theorem method by compressibility. *PeerJ Computer Science*, 1:e23, 2015.
[39] Fernando Soler-Toscano, Hector Zenil, Jean-Paul Delahaye, and Nicolas Gauvrit. Calculating Kolmogorov complexity from the output frequency distributions of small Turing machines. *PloS one*, 9(5):e96223, 2014.

[40] Hector Zenil, Fernando Soler-Toscano, Kamaludin Dingle, and Ard A Louis. Correlation of automorphism group size and topological properties with program-size complexity evaluations of graphs and complex networks. *Physica A: Statistical Mechanics and its Applications*, 404:341–358, 2014.
[41] Kamaludin Dingle, Steffen Schaper, and Ard A Louis. The structure of the genotype–phenotype map strongly constrains the evolution of non-coding RNA. *Interface focus*, 5(6):20150053, 2015.
[42] J.M.G. Vilar, H.Y. Kueh, N. Barkai, and S. Leibler. Mechanisms of noise-resistance in genetic oscillators. *Proceedings of the National Academy of Sciences of the United States of America*, 99(9):5988, 2002.
[43] K. Willbrand, F. Radvanyi, J.P. Nadal, J.P. Thiery, and T.M.A. Fink. Identifying genes from up–down properties of microarray expression series. *Bioinformatics*, 21(20):3859–3864, 2005.
[44] T.M.A. Fink, K. Willbrand, and F.C.S. Brown. 1-d random landscapes and non-random data series. *EPL (Europhysics Letters)*, 79(3):38006, 2007.
[45] K.C. Chen, L. Calzone, A. Csikasz-Nagy, F.R. Cross, B. Novak, and J.J. Tyson. Integrative analysis of cell cycle control in budding yeast. *Molecular Biology of the Cell*, 15(8):3841, 2004.
[46] Geoffrey Grimmett and David Stirzaker. *Probability and random processes*. Oxford University Press, 2001.
[47] Terence Tao, Van Vu, Manjunath Krishnapur, et al. Random matrices: universality of ESDs and the circular law. *The Annals of Probability*, 38(5):2023–2065, 2010.
[48] Alan Edelman and Eric Kostlan. How many zeros of a random polynomial are real? *Bulletin of the American Mathematical Society*, 32(1):1–37, 1995.
[49] E. Borenstein and D.C. Krakauer. An end to endless forms: epistasis, phenotype distribution bias, and nonuniform evolution. *PLoS Comput Biol*, 4(10):e1000202, 2008.
[50] M. Mezard and A. Montanari. *Information, physics, and computation*. Oxford University Press, USA, 2009.
[51] Robert M Gray. *Toeplitz and circulant matrices: A review*. Now Publishers Inc, 2006.
